# *Pf*GCN5 is essential for *Plasmodium falciparum* survival and transmission and regulates Pf H2B.Z acetylation and chromatin structure

**DOI:** 10.1093/nar/gkaf218

**Published:** 2025-03-29

**Authors:** Jingyi Tang, Lee M Yeoh, Myriam D Grotz, Christopher D Goodman, Scott A Chisholm, Hanh H T Nguyen, Chunhao Yu, Kapil Pareek, Fairley McPherson, Anton Cozijnsen, Samuel A Hustadt, Gabrielle A Josling, Karen P Day, Danae Schulz, Geoffrey I McFadden, Tania F de Koning-Ward, Michaela Petter, Michael F Duffy

**Affiliations:** School of Medicine, Deakin University, Waurn Ponds, Victoria 3216, Australia; Institute for Mental and Physical Health and Clinical Translation, Deakin University, Geelong, Victoria 3220, Australia; Department of Microbiology and Immunology, Peter Doherty Institute for Infection and Immunity, The University of Melbourne, Melbourne, Victoria 3000, Australia; Department of Life Sciences, Macfarlane Burnet Institute for Medical Research and Public Health, Melbourne, Victoria 3004, Australia; Mikrobiologisches Institut – Klinische Mikrobiologie, Immunologie und Hygiene, Universitätsklinikum Erlangen, Friedrich–Alexander–Universität (FAU) Erlangen–Nürnberg, 91054 Erlangen, Germany; School of BioSciences, The University of Melbourne, Parkville, Victoria 3052, Australia; School of BioSciences, The University of Melbourne, Parkville, Victoria 3052, Australia; Bio21 Institute, 30 Flemington Road Parkville, Victoria 3052, Australia; Bio21 Institute, 30 Flemington Road Parkville, Victoria 3052, Australia; Department of Medicine, The Royal Melbourne Hospital, The University of Melbourne, Parkville, Victoria 3052, Australia; Mikrobiologisches Institut – Klinische Mikrobiologie, Immunologie und Hygiene, Universitätsklinikum Erlangen, Friedrich–Alexander–Universität (FAU) Erlangen–Nürnberg, 91054 Erlangen, Germany; Mikrobiologisches Institut – Klinische Mikrobiologie, Immunologie und Hygiene, Universitätsklinikum Erlangen, Friedrich–Alexander–Universität (FAU) Erlangen–Nürnberg, 91054 Erlangen, Germany; Department of Microbiology and Immunology, Peter Doherty Institute for Infection and Immunity, The University of Melbourne, Melbourne, Victoria 3000, Australia; Bio21 Institute, 30 Flemington Road Parkville, Victoria 3052, Australia; School of BioSciences, The University of Melbourne, Parkville, Victoria 3052, Australia; Mikrobiologisches Institut – Klinische Mikrobiologie, Immunologie und Hygiene, Universitätsklinikum Erlangen, Friedrich–Alexander–Universität (FAU) Erlangen–Nürnberg, 91054 Erlangen, Germany; Department of Medicine, The Royal Melbourne Hospital, The University of Melbourne, Parkville, Victoria 3052, Australia; Department of Microbiology and Immunology, Peter Doherty Institute for Infection and Immunity, The University of Melbourne, Melbourne, Victoria 3000, Australia; Bio21 Institute, 30 Flemington Road Parkville, Victoria 3052, Australia; The Department of Biology, Harvey Mudd College, Claremont, CA 91711, United States; School of BioSciences, The University of Melbourne, Parkville, Victoria 3052, Australia; School of Medicine, Deakin University, Waurn Ponds, Victoria 3216, Australia; Institute for Mental and Physical Health and Clinical Translation, Deakin University, Geelong, Victoria 3220, Australia; Department of Microbiology and Immunology, Peter Doherty Institute for Infection and Immunity, The University of Melbourne, Melbourne, Victoria 3000, Australia; Mikrobiologisches Institut – Klinische Mikrobiologie, Immunologie und Hygiene, Universitätsklinikum Erlangen, Friedrich–Alexander–Universität (FAU) Erlangen–Nürnberg, 91054 Erlangen, Germany; Department of Microbiology and Immunology, Peter Doherty Institute for Infection and Immunity, The University of Melbourne, Melbourne, Victoria 3000, Australia; Bio21 Institute, 30 Flemington Road Parkville, Victoria 3052, Australia

## Abstract

*Plasmodium falciparum* causes most malaria deaths. Its developmental transitions and environmental adaptation are partially regulated by epigenetic mechanisms. *Plasmodium falciparum* GCN5 (*Pf*GCN5) is an epigenetic regulator that acetylates lysines and can also bind to acetylated lysine residues on histones via its bromodomain (BRD). Here, we showed that *Pf*GCN5 was essential for parasite transmission and survival in human blood and mosquitoes. *Pf*GCN5 regulated genes important for metabolism and development and its BRD was required at euchromatic gene promoters for their proper expression and for acetylation of the variant histone Pf H2B.Z. However, *Pf*GCN5 was most abundant in heterochromatin and loss of the *Pf*GCN5 BRD de-repressed heterochromatic genes and increased levels of acetylated Pf H2B.Z in heterochromatin. The *Pf*GCN5 BRD-binding compound L-45 phenocopied deletion of the *Pf*GCN5 BRD, identifying *Pf*GCN5 as a promising drug target for BRD inhibitors. Thus, *Pf*GCN5 appears to directly contribute to activating euchromatic promoters, but *Pf*GCN5 is also critical for maintaining repressive heterochromatin structure.

## Graphical Abstract

**Graphical Abstract:**
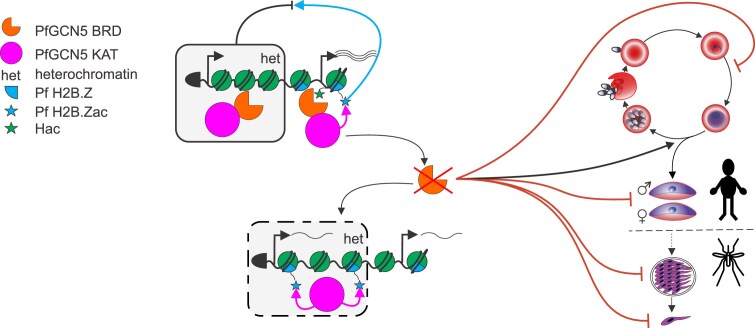

## Introduction

*Plasmodium falciparum* differentiates to survive in diverse environments in mammalian and mosquito hosts. It also must adapt to genetic differences within a host species and to changes within a single host, e.g. development of immunity. The parasite uses epigenetics to regulate these adaptations [1–3]. The parasite can transduce environmental signals, e.g. lysophosphatidylcholine depletion in plasma triggers parasite development into transmissible gametocytes [4]. However, *P. falciparum* largely relies on a bet-hedging strategy for adaptation during its 48-h asexual, intraerythrocytic developmental cycle. It achieves this by occasionally switching expression between members of multigene families that are otherwise silenced in facultative heterochromatin [5]. This strategy enables the intraerythrocytic parasites to evade the host immune response by antigenic variation and switch to use of different cytoadhesion receptors, soluble nutrient transporters, and erythrocyte invasion receptors (reviewed in [6]).

*P. falciparum* silences genes in heterochromatin by histone deacetylation, partially catalyzed by PfSir2A and B, trimethylation of H3K9, and binding of heterochromatin protein 1 [7–11]. Conversely euchromatic active genes carry multiple acetylations of H3 [9, 12], H4 [13], Pf H2A.Z, and Pf H2B.Z [14]. It is unclear which enzyme is responsible for which histone post-translational modification. GCN5 is a lysine acetyltransferase that in other eukaryotes acts as part of complexes that are associated with gene activation in euchromatin, e.g. the SAGA complex that recruits the general transcriptional machinery to promoters [15]. GCN5 contains a bromodomain (BRD) that can bind acetylated lysines on histones and recruits GCN5-containing complexes to chromatin where GCN5 can further acetylate histone lysines.

The *P. falciparum* ortholog of GCN5 (*Pf*GCN5) was implicated in activating erythrocyte invasion genes, suppressing gametocytogenesis [16] and in regulating stress response genes [17–19]. *Pf*GCN5 is part of a SAGA-like complex along with the GCN5 coactivator ADA2 and *Pf*PHD1 and *Pf*PHD2 that carry methylated lysine-binding plant homeodomain (PHD) domains [16, 20]. Recruitment of *Pf*GCN5 to *P. falciparum* promoters activates genes [21, 22] and loss of the *Pf*GCN5 BRD was previously reported to disrupt gene expression and result in de-repression of some heterochromatic genes by an unknown mechanism [16].

In yeast, GCN5 can partially acetylate the alternative histone H2A.Z [23] which is deposited by the Swr1 complex [24]. The spread of telomeric heterochromatin by the yeast Sir2 histone deacetylase is antagonized by both H2A.Z [25] and GCN5 [26]. In *P. falciparum, Pf*GCN5 is associated with Swr1 [20], and Pf H2A.Z exclusively associates with the apicomplexan-specific variant histone Pf H2B.Z [27]. In asexual blood stages, Pf H2A.Z and Pf H2B.Z are excluded from heterochromatin by PfSir2A and B [14], but in female gametocytes Pf H2A.Z and Pf H2B.Z are dramatically enriched in heterochromatin [28], suggesting that the variant histones play an important and dynamic role in organizing heterochromatin structure in *P. falciparum* during development.

Here, we investigated the function of *Pf*GCN5 in regulating parasite development and in maintenance of chromatin structure. By inducible knockout [29] and knock sideways [30] of *Pf*GCN5, we show that *Pf*GCN5 is critical for survival of blood stage asexual and sexual parasites and transmission to mosquitoes. Chromatin immunoprecipitation sequencing (ChIPseq) and RNA sequencing (RNAseq) reveal that *Pf*GCN5 at euchromatic promoters contributes to their activation and *Pf*GCN5 is enriched at genes important for developmental transitions. Strikingly, *Pf*GCN5 also maintains silent heterochromatin through regulating the dynamics of the alternative histone Pf H2B.Z and its acetylation.

## Materials and methods

### Parasite culture

Blood-stage *P. falciparum* were grown in RPMI–HEPES supplemented with 10% human serum or 0.5% AlbuMAX^®^ II Lipid-Rich BSA (Life Technologies, 11021), 0.2% sodium bicarbonate, and human O^+^ red blood cells (RBCs) at 4% haematocrit. Parasites were incubated at 37°C in 5% CO_2_, 1% O_2_, and 94% N_2_. Ring-stage parasites were synchronized to a ∼4-h window by incubations with 5% D-sorbitol separated by 12 h. The wild-type (WT) 3D7 parental line was maintained without the addition of drug, whereas integrated transgenic cell lines 3D7-(Pfgcn5::TY1) and 3D7-(Pfgcn5::Fkbp::Gfp) were grown in the presence of 4 nM WR99210 and NF54::diCre-(Pfgcn5:loxP) in the presence of 20 nM WR99210 (Jacobus Pharmaceuticals). 3D7-(Pfgcn5::Fkbp::Gfp) were also grown with 7 μg/ml blasticidin S hydrochloride (Sigma–Aldrich, 15205) to maintain the episomal pLyn-FRB-mCherry. Knock sideways by mislocalization of PFGCN5::FKBP::GFP to the cytoplasm was achieved by dimerization with Lyn::FRB::mCherry in the presence of 250 nM rapalog (A/C heterodimerizer, 635056, Takara) [30].

For gametocyte induction, parasites were tightly synchronized and cultured in partially spent medium as described previously [28]. Briefly, cultures were prepared at 1.5% trophozoite parasitemia and 5% haematocrit on day −3. On day −2, the haematocrit was lowered to ∼3% by addition of fresh medium. On day −1, the cultures were split to 1.5% parasitemia at 5% haematocrit and allowed to reinvade overnight. From day 0 onwards, when asexual and sexual rings were present, the culture medium was changed daily. Addition of 20 U/ml of heparin (Sigma–Aldrich, H3149) from day 0 to day 4 (for commitment assays) or day 5 (maturation assays) suppressed reinvasion of asexual parasites. For commitment assays, BRD excision was induced by addition of 250 nM rapamycin (Santa Cruz) on day −3, when parasites were in the trophozoite stage. For gametocyte maturation assays, Dimethyl sulfoxide (DMSO) or rapamycin were added to the culture on day 0, consisting of asexual and sexual rings. Gametocytes were counted and morphologically evaluated by daily Giemsa smears. The sexual conversion rate was calculated as follows: (% gametocyte-infected RBCs day 4/% ring-infected RBCs day 0) *100%.

For mosquito infections, standard membrane feeding assays were performed as previously [31]. Briefly, parasite cultures were started at 0.5% parasitaemia and 5% haematocrit (Australian Red Cross Blood Service) in RPMI-1640 GlutaMax medium (Gibco) supplemented with heat inactivated (HI) human serum (Australian Red Cross Blood Service) and hypoxanthine (25 mg/ml in 1 M NaOH). Medium was replaced every 24 h on a 37°C heating stage. Knockout of the *pfgcn5* BRD was induced on day 7 of gametocyte development. Thin blood smears with methanol fixation and Giemsa staining [10% (v/v), Sigma–Aldrich] were created to confirm gametocyte development at day 17. On day 18, gametocyte cultures were diluted with washed erythrocytes and mixed with 0.48 ml HI human serum to yield a final concentration of 0.15% stage V gametocytemia in 1.08 ml and added to prewarmed glass feeders. Adult females of *Anopheles stephensi* (Malaria Research and Resource Reference Centre) were fed in low light for 20 min, then returned to the incubator. Sucrose feeding began 2 days after blood feeding.

To assess exflagellations, 3 μl of day 18 gametocyte culture was added to 100 μl exflagellation media [10% HI fetal bovine serum and RPMI-1640 GlutaMax (pH 8.4)] and incubated for 15–20 min at 22°C. Exflagellations and erythrocytes were counted using an Olympus CX43 compound light microscope. To assess oocyst numbers, mosquito midguts were removed 7 days after infection, stained in 0.4% mercurochrome, and oocysts counted on an Olympus CX43 compound light microscope. To assess sporozoite numbers, mosquito salivary glands were removed 20–21 days after infection, homogenized in a plastic grinder, and diluted appropriately to count using a hemocytometer. Sporozoites were observed on an Olympus CK2 inverted light microscopy (at 20 times magnification).

### Plasmid DNA cloning

To make the plasmid pTgcn5:TY1, the three HA tags in pTHA [27] were excised with *Nhe*I and *Spe*I and replaced with 3xTY1 epitope tags to make pTTY1 and then a 718-bp fragment from the C-terminal end of the *pfgcn5* gene was amplified with primers TTTcccgggCAAAAGAAAGA and GgctagcTTCTTTTGCTGTATCAG and cloned into the plasmid pTTY1 using the restriction enzymes *Xma*I and *Nhe*I (Supplementary Fig. S1A). To create the pSLI-GCN5-sandwich construct, the 1048-bp C-terminal DNA sequence from nucleotide position 3713 to 4760 of the *Pf*GCN5 gene (PF3D7_0823300) without the stop codon was amplified by polymerase chain reaction (PCR). The resulting product was digested with the restriction enzymes *Not*I and *AvrI*I and cloned into the corresponding sites of the pSLI-sandwich plasmid (pSLI-2xFKBP-GFP-2xFKBP) [32] (Supplementary Fig. S2A). To make the Pfgcn5:loxP plasmid, the vector backbone (a kind gift from Simon Cobbold, Danushka Marapana, and Alan Cowman) and cloning strategy of Wilde *et al.* [33] were used. Briefly, the last 360 bp of the *pfgcn5* gene (nucleotide positions 4039–4398) containing the BRD were recodonized and flanked by loxP sites. The loxP site upstream was contained in a synthetic intron, with the coding sequence of the gene followed by three haemagglutinin tags, three stop codons, a loxP site, linker, *glmS* switch, and selectable marker (human dihydrofolate reductase cassette) (Supplementary Fig. S3A and B).

### Parasite transfection and generation of parasite lines

3D7-(Pfgcn5::TY1) were made from 3D7 parasites by integrating 3 × TY1 epitope tags 3′ of the *Pf*GCN5 coding sequence. In brief, 100 μg of pTgcn5:TY1 plasmid DNA in 15 μl warm tris ethylenediaminetetraacetic acid (TE) buffer was mixed with 385 μl of warm cytomix [120 mM KCl, 0.15 mM CaCl_2_, 2 mM ethylene glycol tetraacetic acid (EGTA), 5 mM MgCl_2_, 10 mM K_2_HPO_4_/KH_2_PO_4_ (pH 7.6), 25 mM HEPES (pH 7.6)], before adding to 180 μl of fresh RBCs that were pre-washed in cytomix. The mixture was then electroporated. After washing in RPMI–HEPES, DNA-loaded RBCs were added to 2 × 10^7^ synchronized trophozoites and cultured in the presence of 2.5 nM of WR99210. Parasites surviving drug pressure were then grown in the absence of WR99210 for 3 weeks to remove episomes after which drug selection was reapplied to select for integrants. 3D7-(Pfgcn5::TY1) clones were obtained by limiting dilution.

3D7-(Pfgcn5::Fkbp::Gfp) were made from 3D7 parasites by integrating the pSLI-GCN5-sandwich plasmid in-frame and 3′ of *Pf*GCN5. pSLI-GCN5-sandwich contained two FKBP dimerization domains followed by green fluorescent protein (GFP) and then two more FKBP dimerization domains then the 2A skip peptide and neomycin resistance gene and human *dhfr* cassette required for selection-linked integration [30] (Supplementary Fig. S2). Synchronized ring-stage 3D7 parasites were transfected with 100 μg of pSLI-GCN5-sandwich plasmid DNA. Briefly, 370 μl of warm cytomix (pH 7.6) was added to plasmid DNA in 30 μl TE, mixed gently with 1 × 10^8^ of ring-stage parasites, transferred to a 2-mm gap cuvette, and electroporated at conditions of 310 V, 950 μF, and infinite resistance using a Bio-Rad Gene Pulser. Parasites were initially selected with WR99210 at 4 nM and parasites surviving drug pressure were subsequently selected with neomycin (Sigma–Aldrich, G-418, 4727878001) at 400 μg/ml as previously described [30, 34]. A clonal population of parasites containing the integrated pSLI-GCN5-sandwich plasmid was obtained by fluorescence-activated cell sorting of parasites expressing GFP using a BD FACSArial III cell sorter and integration was confirmed by PCR (Supplementary Fig. S2A and B). The clonal population was then transfected [30] as described above with the plasma-membrane mislocalizer plasmid encoding the lyn N-terminal plasma membrane localization peptide, the mammalian target of rapamycin (mTOR) FRB dimerization domain, and the mCherry fluorescent protein gene (pLyn-FRB-mCherry) to generate 3D7-(Pfgcn5::Fkbp::Gfp) parasites. The lyn::FRB::mCherry fusion protein could be induced to dimerize with *Pf*GCN5::FKBP::GFP by the addition of rapalog, thereby mistargeting *Pf*GCN5 to the parasite plasma membrane [30].

NF54::diCre-(Pfgcn5:loxP) parasites were made from NF54::DiCre parasites that expressed Cre recombinase from an integrated trans gene [29] by integrating the Pfgcn5:loxP plasmid into the *Pfgcn5* locus. This introduced *loxP* sites in artificial introns each side of the *Pfgcn5* BRD and fused *Pfgcn5* to three C-terminal HA tags (Supplementary Fig. S3A). Briefly, 100 μg plasmid DNA was linearized by digestion with *Bgl*II and *Pvu*I overnight and then transfected along with 100 μg of single guide RNA (sgRNA) plasmid into NF54::DiCre ring-stage parasites using the same conditions described above for transfection of 3D7-(Pfgcn5::Fkbp::Gfp). Transfected parasites were resuspended in 4% haematocrit erythrocytes and one day after transfection drug selection was started using 20 nM WR99210.

For all cell lines, integrants were cloned and confirmed by PCR and/or sequencing (Supplementary Figs S1, S3A–C, and S2A and B).

### Live cell imaging of 3D7-(Pfgcn5::Fkbp::Gfp) and immunofluorescence of 3D7-(Pfgcn5::TY1)

For live cell imaging, Hoechst 33342 (ThermoFisher) was added to 1 × 10^7^ parasites in phosphate-buffered saline (PBS) at a final concentration of 2 μg/ml. After 5 min incubation on a roller in the dark, the infected RBCs were washed three times in PBS, and the pellet obtained after centrifugation at 1500 × *g* was resuspended in 1–1.5× pellet volume of PBS. Then, 5 μl was loaded on a glass microscope slide and covered with a #1.5 coverslip. Imaging was acquired using a Deltavision Elite widefield deconvolution fluorescence microscope with a CCD camera and a 100× oil objective. Images were subjected to deconvolution using the default settings on the software SoftWoRx 6.1 and processed using Fiji ImageJ (version 2.1.0/1.53c). For immunofluorescence, cells were fixed with paraformaldehyde/glutaraldehyde and adhered to glass slides and permeabilized as previously described [35]. Cells were probed with mouse anti-Ty1 antibody (Sigma–Aldrich, SAB4800032-50UG) and bound anti-Ty1 antibody detected with goat anti-mouse IgG-Alexafluor 488. DNA was stained with Hoechst 34580. Imaging was acquired as per the live cell imaging.

### Immunofluorescence assay of gametocytes

Immunofluorescence assays of gametocyte experiments were conducted on dried blood smears fixed with ice-cold 10% methanol/90% acetone. Using a silicon pen (DakoCytomation), different fields were drawn on the smear. After rehydrating in PBS for 10 min, the primary antibodies in PBS/3% bovine serum albumin (BSA) were added to each field and incubated for 2 h at room temperature in a moist box. After three washes with PBS, secondary antibody and Hoechst were added in PBS/3% BSA for 2 h. After three more washing steps with PBS, the slides were mounted with a drop of anti-fading medium (Mowiol with Dabco) before covering with a coverslip. The mounted slides were cured overnight and analysed using a 100× oil objective fluorescence microscope using a Zeiss Axio Observer microscope.

### Parasite growth assays

For the 7-day growth assay by flow cytometry and the validation of knock sideways of *Pf*GCN5, 3D7-(Pfgcn5::Fkbp::Gfp) parasites and 3D7 WT control parasites were synchronized with sorbitol into a ∼4-h window. On day 0, ring stages were diluted to 0.1% parasitemia for growth assays or 5% parasitemia to validate knock sideways with the addition of 250 nM rapalog or vehicle (end ethanol concentration was 0.05%). Successful knock sideways of *Pf*GCN5 was validated by live cell imaging of 3D7-(Pfgcn5::Fkbp::Gfp) at 5% parasitemia on day 1, ∼24 h after the treatment with rapalog/vehicle. The vehicle-treated parasites starting at 0.1% parasitemia and the 3D7 control parasites treated with rapalog were all split one-tenth at the end of day 5 to avoid overgrowth.

For flow cytometry from day 1–7, 3D7-(Pfgcn5::Fkbp::Gfp) parasites treated with rapalog or vehicle were freshly stained with both 2 μM Hoechst 34580 (ThermoFisher, H21486) and 1 μM SYTO61 (Invitrogen). Cells were washed and suspended to 0.5% haematocrit in RPMI–HEPES and triplicate 50 μl aliquots were stained for 30 min in the dark then washed twice and finally resuspended in 200 μl RPMI–HEPES. To determine voltage and gating parameters for flow cytometry, synchronized 3D7 parasites at 5% parasitemia were used either unstained, stained with Hoechst 34580 only, SYTO61 only, or both, and double-stained fresh uninfected RBCs were used as negative controls. All samples were analysed by the FACSCanto II (BD Biosciences) flow cytometer in high-throughput mode at slow flow rate with 500 000 RBCs as the target events for each well, based on the forward versus side scatter profiles. Hoechst 34580 fluorescence was excited using a violet laser at 405 nm and measured with a 450/40 nm filter. SYTO61 fluorescence was excited using a red laser at 633 nm and measured with a 660/20 nm filtre. Variations in the detector gain settings were kept minimal across the 7 days and constant within each day, and no compensation was applied to each channel. Data analyses were performed using FlowJo 10.7.2 software. The growth assay by flow cytometry was performed in three biological replicates.

To assess parasite growth by Giemsa-stained smears, synchronized ring-stage 3D7-(Pfgcn5::Fkbp::Gfp) were diluted to 1.5% parasitemia on day 0 with the addition of 250 nM rapalog or vehicle (final ethanol concentration was 0.05%). The vehicle group was split one-fifth at the end of days 3 and 5; and one-half at the end of day 6. The rapalog-treated group was split one-half at the end of both, days 3 and 5. Growth characteristics and cell morphology were monitored for 7 days by microscopy of Giemsa-stained blood smears that had been fixed with methanol.

The growth of NF54::diCre-(Pfgcn5:loxP) parasites was assessed by Giemsa-stained microscopy, SYBR assay [36], and flow cytometry. For biological triplicate flow cytometry, parasites were synchronized with sorbitol into a ∼4-h window, then diluted as appropriate to prevent overgrowth, and grown in 96-well round-bottom plates in technical triplicates. After the required duration of growth, parasites were harvested by centrifugation, medium was aspirated, then parasites were washed in PBS twice and stored in PBS up to 12 h at 4°C in the dark. Parasites were later stained for 30 min in the dark in 1 μM SYTO 61 and 1 μg/ml thiazole orange. Parasites were washed twice with PBS, then immediately read on a BD FACS Canto II. The parasites were analysed by cytometry and Giemsa-stained microscopy for all timepoints up to 112 h. For subsequent timepoints, parasites were analysed by Giemsa-stained microscopy only. To compare growth, ratios of parasitemias were multiplied by the dilution factors used to set up the assays.

SYTO61 stains both DNA and RNA, whereas Hoechst 34580 only stains DNA and thiazole orange only stains RNA. The staining of both DNA and RNA is proposed to better separate the different populations by stage. Single stainings were used for compensation of the fluorescent activated cell sorting (FACS) settings and double stainings were used to gate the cell populations in both experiments.

Growth inhibition of 3D7 strain *P. falciparum* by L-45 was assessed in biological and technical triplicate in 96-well plates. Each plate included L-45-treated erythrocyte-only controls, the other wells contained 0.1 ml aliquots of 4% haematocrit erythrocytes infected with 3D7 strain *P. falciparum* at 0.5% parasitemia. Infected erythrocytes were treated with DMSO as a vehicle-only control or with dilutions of L-45. Plated parasites were grown at 37°C for 72 h in 5% CO_2_, 1% O_2_, and 94% N_2_. After 72 h, parasitized erythrocytes were lysed with 0.08% (v/v) Triton X-100 and 0.008% saponin in 20 mM Tris–HCl, 5 mM ethylenediaminetetraacetic acid (EDTA; pH 7.5), and DNA stained with SYBR green for 1 h at room temperature prior to quantitating fluorescence using a BMG CLARIOstar Plus. Background (uninfected erythrocytes) was subtracted from fluorescence readings, and relative growth calculated by dividing by a vehicle-only control.

### Parasite cell fractionation

Infected erythrocytes were washed with PBS and the erythrocytes lysed by incubation in ice-cold 0.075% saponin/PBS for 5 min. Parasites were pelleted by centrifugation at 2500 × *g* for 5 min and washed twice with 10 ml of ice-cold PBS. For total protein extracts, the parasites were then suspended in 2× Laemmli buffer and boiled at 95°C for 10 min, subjected to centrifugation at 2500 × *g* for 10 min and the supernatant containing the total protein extract was aspirated. For cellular fraction, the saponin-released parasites were incubated on ice with 500 μl lysis buffer [20 mM HEPES (pH 7.8), 10 mM KCl, 1 mM EDTA, 1 mM DL-dithiothreitol (DTT), 1× protease inhibitors (Roche, catalogue no. 4693159001)] for 15 min and then the parasite cell membranes were selectively lysed by incubation with 0.65% (v/v) IGEPAL 630 (Sigma, catalogue no. I8896) on ice for 1 min. The lysed cells were subjected to centrifugation at 2500 × *g* for 10 min at 4°C and cytoplasmic proteins were aspirated in the supernatant. The pelleted nuclei were washed with lysis buffer and then vigorously agitated in 800 mM KCl nuclear extraction buffer [20 mM HEPES (pH 7.8), 800 mM KCl, 1 mM EDTA, 1 mM DTT, 1× protease inhibitors) for 30 min at 4°C, then centrifuged at 2500 × *g* for 30 min and soluble nuclear proteins were aspirated in the supernatant. The pellet was dissolved by boiling in 2× Laemmli buffer and then centrifuged at 8000 × *g* for 10 min and the supernatant containing histones and other proteins tightly associated with chromatin was diluted in 20 mM HEPES, 1 mM DTT, 1 mM EDTA, and 30% glycerol. All fractions were analysed by western blot.

### Native chromatin immunoprecipitation

Chromatin was isolated from late trophozoite-stage (30–38 hpi) *Pf*GCN5-Ty or *Pf*3D7 WT parasites. Briefly, 0.96–1.2 $ \times$ 10^9^ infected RBCs were lysed with cold saponin and incubated with 500 μl cold lysis buffer [10 mM HEPES (pH 7.9), 10 mM KCl, 0.1 mM EDTA, 0.1 mM EGTA, 1 mM DTT, 1$ \times$ EDTA-free protease inhibitor (Roche, 04693132001), 10 mM sodium butyrate] for 15 min on ice. IGEPAL^®^ CA-630 (Sigma–Aldrich, I8896) was then added to a final concentration of 0.65% and incubated for 1 min on ice. The nuclei were pelleted at 2500 × *g* for 10 min at 4°C and the supernatant was saved as the cytoplasm fraction for analysis. The nuclei were washed once in cold lysis buffer before being resuspended in 200 μl cold chromatin digestion buffer [50 mM Tris (pH 7.4), 4 mM MgCl_2_, 1 mM CaCl_2_, 1× EDTA-free protease inhibitor, 5 mM sodium butyrate]. Then, 1 μl of micrococcal nuclease (MNase; NEB, M0247S) diluted one-tenth was added and the mixture was incubated at 37°C for 15 min. EDTA was then added to a final concentration of 10 mM to stop the reaction. The nuclei were pelleted at 2500 × *g* for 10 min at 4°C and the supernatant was saved as the MNase soluble fraction. The nuclei were further extracted with 200 μl cold high-KCl buffer [20 mM HEPES (pH 7.9), 800 mM KCl, 1 mM EDTA, 1 mM DTT, and 1× EDTA-free protease inhibitor] at 4°C spinning for 1 h on a roller. The nuclei were pelleted at 2500 × *g* for 30 min at 4°C and the supernatant was saved as the high salt-soluble fraction. Finally, the nuclei were extracted with 200 μl cold sodium dodecyl sulfate (SDS) extraction buffer [10 mM Tris (pH 8.0), 2% SDS] at 4°C spinning for 30 min. The nuclei were pelleted at 13 000 rpm for 5 min at 4°C and the supernatant was saved as the SDS-soluble fraction. All fractions were stored at 4°C and used within 1 week.

Chromatin immunoprecipitation (ChIP) was carried out immediately on the day following chromatin harvest. The MNase-soluble fraction was diluted 1:7 in RIPA buffer [1% IGEPAL^®^ CA-630, 150 mM NaCl, 50 mM Tris–HCl (pH 7.4), 1 mM EDTA, 1× EDTA-free protease inhibitor]. Diluted chromatin was pre-cleared using equilibrated protein A/G beads (GE Healthcare, 17-5280-01 and 17-0618-01) at a packed beads-to-chromatin volume ratio of 1:70 for 1 h at 4°C. Then, 600 μl pre-cleared chromatin from 4.8–6.0 $ \times$ 10^8^ late trophozoite-stage parasites were immunoprecipitated overnight rotating at 4°C with 25 μl pre-washed packed protein A/G beads and 5 μg mouse anti-Ty1 antibody (Sigma–Aldrich, SAB4800032-50UG) or mouse IgG control antibody (0.5mg/ml). One-tenth of the volume of immunoprecipitated chromatin (60 μl) was also incubated overnight at 4°C for use as the input control. Beads were washed six times with RIPA buffer, each for 10 min at 4°C rotating, and spun down at 12 000 × *g* for 1 min at 4°C between wash. Co-immunoprecipitated DNA was eluted twice with 100 μl freshly made elution buffer (1% SDS, 100 mM NaHCO_3_) and purified using the MinElute PCR Purification kit (Qiagen, catalogue no. 28006). ChIP experiments were performed in two biological replicates.

### Cross-linked histone ChIP

NF54::diCre-(Pfgcn5:loxP) cultures were synchronized to a 6-h developmental window using Percoll enrichment of schizont-stage parasites followed by sorbitol synchronization 6 h later, treated with rapamycin or DMSO at ring stage (10–16 hpi), and analysed after 72 h (34–40 hpi) by immunoblot and ChIPseq in duplicates. For ChIPseq, cultures were fixed using 1% paraformaldehyde and chromatin was prepared using standard procedures [28]. Immunoprecipitation was conducted in low-bind tubes (Eppendorf) over night at 4°C using antibodies directed against Pf H2B.Z and Pf H2B.Zac [14] and further processed as described before [28]. Precipitated DNA was purified using the MinElute kit (Qiagen) and quantified using a Qubit HS assay (Life Technologies).

### Histone acetylation western blots

Protein extracts from saponin-lysed parasites [NF54::diCre-(Pfgcn5:loxP) or NF54 treated for 4 h with L-45] were separated on 12% Bis–Tris Gels in 1× MES buffer (Life Technologies) and transferred to nitrocellulose membranes. After 30 min blocking in TBS-Tween (TBS-T) with 5% non-fat dried milk powder, primary antibodies were added and incubated over night at 4°C. Following three washes in TBS-T, horseradish peroxidase-coupled secondary antibodies were added for 2 h at room temperature. After three more washes, the proteins were detected with Immobilon ECL reagent (Millipore) and images were acquired using the Vilber Fusion FX System. Densitometry was performed using ImageJ [37].

### ChIPed DNA library preparation for sequencing

ChIPed DNA libraries were prepared using the NEBNext^®^ Ultra^TM^ II DNA library prep kit for Illumina^®^ (New England BioLabs, E7645S) or the Accel-NGS™ 2S Plus DNA Library Kit for Illumina platforms (SWI Swift Bioscience) and the Accel-NGS™ 2S Indexing Kit (SWI Swift Bioscience). In brief, 4 ng input or chromatin-immunoprecipitated DNA was used and adaptor-ligated DNA was purified without size selection using Agencourt AMPure XP beads (Beckman Coulter, A63881). The PCR amplification step was performed using 1 unit of high-fidelity KAPA HiFi DNA polymerase (KAPA biosystems, KK2101), 0.3 mM dNTPs, 1 μM NEBNext i5 primer, and 1 μM NEBNext i7 primer [NEBNext^®^ Multiplex Oligos for Illumina^®^ (Dual Index Primers Set 1), E7600S] in 1$ \times$ KAPA HiFi buffer containing tetramethylammonium chloride for 12 PCR cycles . The PCR conditions were 98°C for 2 min for the initial denaturation followed by 12 cycles of 98°C for 15 s, 65°C for 2 min, and a final extension at 65°C for 5 min. Library clean-up was performed using 0.9$ \times$ AMPure beads and DNA libraries were eluted with low-EDTA TE [10 mM Tris–HCl (pH 8.0), 0.1 mM EDTA (pH 8.0)].

### RNA extraction and RNA library preparation for sequencing

Triplicate RNAs from *Pfgcn5*:ΔBRD and controls and stage-matched RNAs from the two biological replicates used for GCN5::TY1 native ChIP were extracted as previously described [12]. Briefly, the aqueous phase from cells dissolved in TRIzol^®^ reagent (Life Technologies, 15596018) was diluted in 70% ethanol and purified using RNeasy Mini columns (Qiagen, catalogue no. 74104). Contaminating genomic DNA (gDNA) was digested with DNaseI (RNase-Free DNase Set Qiagen, catalogue no. 79254) and RNA integrity assessed by TapeStation (Agilent). Total RNA was used to prepare TruSeq stranded messenger RNA (mRNA) libraries by the Victorian Clinical Genetics Services (VCGS).

### High-throughput sequencing and bioinformatics

Input and chromatin-immunoprecipitated DNA libraries of 3D7-(Pfgcn5::TY1) were 150-bp paired-end sequenced on an Illumina NextSeq 500 at VCGS. mRNA libraries were 150-bp paired-end sequenced on an Illumina NovaSeq 6000 at VCGS. Sequencing quality was assessed by FastQC, and TrimGalore [38, 39] was used to trim read ends with a PHRED quality score threshold of 33 and to remove Illumina adapter sequences. STAR [40] was used to map RNAseq libraries to the *P. falciparum* 3D7 genome version 55 [41] (Plasmodb.org).

Bowtie 2 [42] primary alignments were used to avoid multiple alignments of single reads in repetitive heterochromatin whilst retaining information from the redundant heterochromatic multigene families and the accuracy of the enrichment patterns were confirmed by comparing with mapping with stringent filtering for uniquely mapped reads using the Burrows-Wheeler Aligner BWA [43] with the argument -c 1.

For comparison of read depth in heterochromatin and euchromatin in input and anti-Ty ChIP, the approach of Baumgarten *et al.* [44] was used. BAM files from both replicates of primary alignments only were made with Bowtie 2 and were merged using SAMtools merge [45]. The regions of euchromatin and heterochromatin were defined by model-based analysis for ChIPseq (MACS2) [46] called H3K9me3 broad peaks in trophozoite-stage 3D7 parasites carrying a ABC2-GFP reporter [28]. Mosdepth [47] was used to determine average per region read depths for euchromatin and heterochromatin.

MACS2 [46] was used to call peaks separately for the two *Pf*GCN5::TY ChIPseq replicates and significant peaks shared between replicates were identified using multiple sample peak calling (MSPC) [48]. Peaks confirmed by MSPC in either replicate were combined and the closest gene to the summit of each peak was identified by Bedtools closest. RNAseq-mapped reads were assigned to genes using featureCounts [49], which does not count multimapping reads, and differential gene expression analysis was performed using DESeq2 [50] incorporating two factors of unwanted variation from Removal of Unwanted Variation using control genes (RUVg) [51] in the generalized linear model. Principal Components Analysis (PCA) and correlation heatmaps of sequencing data and heatmaps and average profile plots of RNAseq and ChIP/input enrichment were made using deepTools [52]. Sequencing data were transformed and visualized using SAMtools [45], bedtools [53], Python, pandas, and R. Statistical tests were performed using Graphpad Prism, Python, pandas, and R. Pathway enrichment analysis using GO terms was performed using PlasmoDB [41] and GO data were visualized in bubble plots generated with SRplots (https://www.bioinformatics.com.cn/srplot) or plots from REVIGO [54]. In REVIGO plots, the colour code indicated log_10_(adjusted *P*-value) from Fisher’s exact test of over-representation of members of a gene annotation in the query set of genes and the size of the circles reflects the relative number of *P. falciparum* genes in the gene annotation.

For the analysis of the histone H2B.Z and the acetylated form H2B.Zac, input and ChIP libraries were 150-bp paired-end sequenced on an Illumina NovaSeq 6000 at the NGS Core Facility University Hospital in Bonn. ChIPseq data in fastq format was first quality checked using FastQC v0.11.8 and then trimmed using TrimGalore 0.6.4 [38, 39] to remove any low-quality regions or remaining adapters and the reads were mapped to the *P. falciparum* reference genome (PlasmoDB 3D7 version 59) [41] using Bowtie 2 version 2.3.4.3 with default settings for paired-end reads [42]. Data were transformed to BAM format and replicate files were merged using SAMtools 1.0 [45]. Data were transformed to bigwig files and visualized using deepTools 3.1.2 [52] or IGV.

## Results

### *Pf*GCN5 is present in both the nucleus and cytoplasm of asexual blood-stage parasites

**Figure 1.**
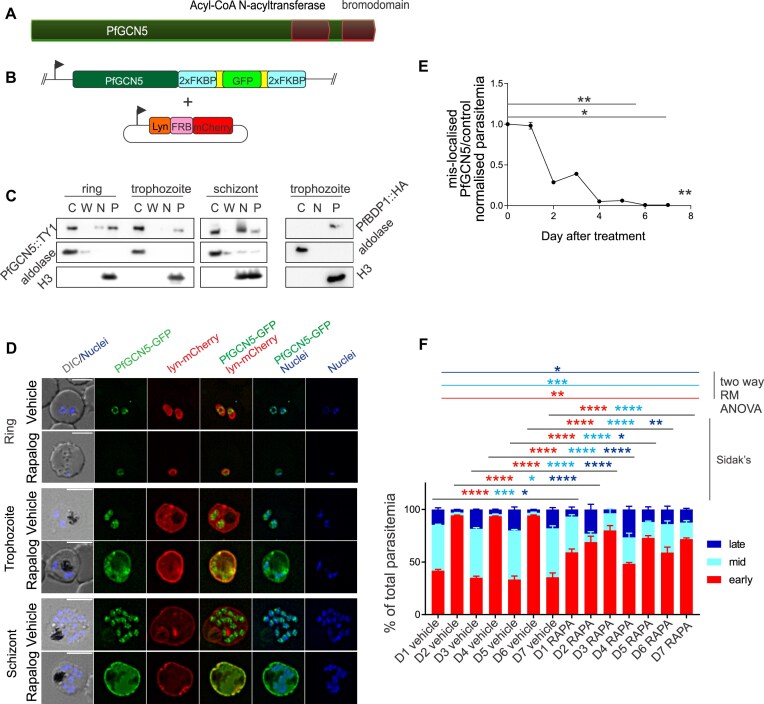
*Pf*GCN5 is present in both nucleus and cytoplasm and is essential for asexual blood-stage parasites. (**A**) Domains in *Pf*GCN5. (**B**) The recombinant *Pf*GCN5 locus in 3D7-(Pfgcn5::Fkbp::Gfp) parasites encoding *Pf*GCN5 fused to FKBP dimerization domains and GFP, and the mislocalizing plasmid encoding the lyn N-terminal plasma membrane localization peptide fused to the mTOR FRB dimerization domain and mCherry. (**C**) Immunoblots of 3D7-(Pfgcn5::TY1) ring stage (<16 hpi), trophozoite (24–34 hpi), and schizont (38–44 hpi) stage cytoplasmic (C), wash (W), 800 mM KCl soluble nuclear (N), and insoluble nuclear (P) fractions probed with anti-TY1, anti-aldolase (cytoplasmic control), and anti-H3 (insoluble nuclear control). PfBDP1::HA [75] trophozoite stage fractionation was conducted in parallel. (**D**) Live fluorescence microscopy of 3D7-(Pfgcn5::Fkbp::Gfp) parasites treated with vehicle or rapalog to induce mislocalization with lyn::FRB::mCherry. Nuclei are stained with Hoechst 33342. Imaging was acquired using a Deltavision Elite widefield deconvolution fluorescence microscope and a 100× oil objective and subjected to deconvolution. DIC: differential interference contrast. Scale bar = 5 μm. (**E**) Normalized parasitemia (parasitemia × dilution factor) measured by flow cytometry and shown as a ratio between 3D7-(Pfgcn5::Fkbp::Gfp) treated with rapalog to mislocalize *Pf*GCN5 over 3D7-(Pfgcn5::Fkbp::Gfp) treated with vehicle alone (*n* = 3 biological replicates, two-tailed Mann–Whitney *U*-test, ****P < 0.0001). (**F**) The relative parasitemias segregated into early-, mid-, and late-stage parasites from gated cytometry data for the three replicates of 3D7-(Pfgcn5::Fkbp::Gfp) for rapalog-induced knock sideways and control over 7 days post-treatment, mean + standard deviation (SD).

**Figure 2.**
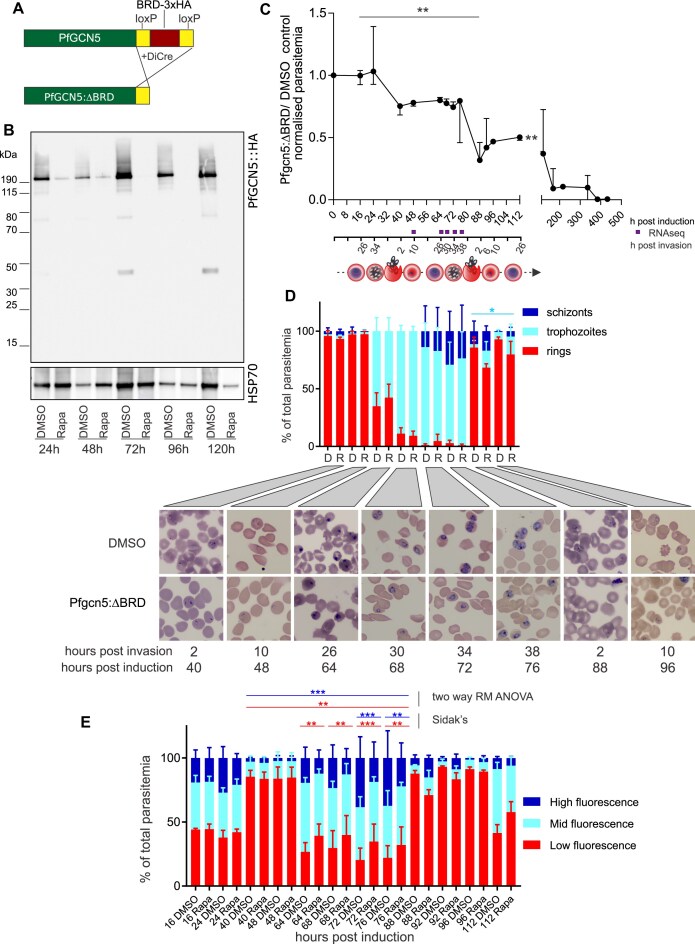
The *Pf*GCN5 BRD is essential and required for asexual blood-stage parasite development. (**A**) The BRD and three HA tags are excised by DiCre in *Pfgcn5*:ΔBRD. (**B**) Immunoblot of *Pfgcn5*:ΔBRD parasites at different times after treatment with DMSO or rapamycin (RAPA) and probed with anti-HA or anti-aldolase as a loading control. (**C**) Ratios of *Pfgcn5*:ΔBRD/control normalized growth (median ± range, *n* = 3 biological replicates). Ratios compared over 0–500 h post-induction (combined cytometry and microscopy) and over 0–112 h (cytometry only) (***P < 0.001, **P < 0.01 Kruskal–Wallis test). (**D**) Giemsa-stained microscopy of parasites from panel (C) with proportion of total parasitemia contributed by each morphological stage indicated (*n* = 3 biological replicates, two-way repeat measures ANOVA, *P < 0.05). (**E**) *Pfgcn5*:ΔBRD cytometry data from samples shown in panel (C) but separated into low, mid, and high fluorescence by gating on nucleic acid staining and presented as the % each population comprised of the total parasites (*n* = 3 biological replicates, two-way repeat measures ANOVA, ***P < 0.001, **P < 0.01).

*Pf*GCN5 is a 174-kDa protein with a histone acetyltransferase domain and a BRD (Fig. 1A). To investigate *Pf*GCN5, we modified the endogenous *Pfgcn5* locus to create three transgenic *P. falciparum* cell lines. For cell fractionation, immunofluorescence, and ChIPseq analyses, we used 3D7-(Pfgcn5::TY1) parasites that expressed a *Pf*GCN5::3xTY1 C terminal epitope-tagged fusion protein (Supplementary Fig. S1A and B). For live cell microscopy and functional studies, we used 3D7-(Pfgcn5::Fkbp::Gfp) that expressed *Pf*GCN5 fused to GFP [30] (Fig. 1B), which could be mislocalized by knock sideways to the parasite plasma membrane by rapalog-induced dimerization with a mCherry fusion protein [30]. For functional studies of the *Pf*GCN5 BRD, we used cloned NF54::diCre-(Pfgcn5:loxP) parasites that expressed a DiCre recombinase that could be induced to excise a loxP-flanked BRD from *Pf*GCN5 fused to three C-terminal HA tags (Fig. 2A and Supplementary S3A) [29].

Full length *Pf*GCN5::TY1 was expressed in both the parasite cytoplasm and nucleus throughout the asexual blood-stage lifecycle as detected by cell fractionation (Fig. 1C and Supplementary Fig. S1C) and immunofluorescence assay (Supplementary Fig. S1D). Within the nucleus, *Pf*GCN5::TY1 was associated with both the 800 mM KCl soluble and the histone-rich insoluble fractions (Fig. 1C). In contrast, the BRD protein PfBDP1 was detected exclusively in the insoluble nuclear fraction (Fig. 1C). This suggests that *Pf*GCN5 may have functions in both nucleus and cytoplasm, as described in other organisms [55]. We note that *Pf*GCN5 was visible only in the nucleus by live fluorescence microscopy of 3D7-(Pfgcn5::Fkbp::Gfp) parasites treated with vehicle alone (Fig. 1D). This differed from the nuclear and cytoplasmic localization of *Pf*GCN5::TY1 in 3D7-(Pfgcn5::TY1) parasites (Fig. 1C and Supplementary Fig. S1C). Possibly the large GFP fusion partner was susceptible to proteolysis in the cytoplasm when un-dimerized in 3D7-(Pfgcn5::Fkbp::Gfp). Cytoplasmic proteolysis of the *Pf*GCN5 N-terminus, has been previously reported [56].

### *Pf*GCN5 is essential for asexual blood-stage *P. falciparum*

To assess the importance of nuclear *Pf*GCN5 for *P. falciparum* growth we performed knock sideways [30] using the synchronized 3D7-(Pfgcn5::Fkbp::Gfp) parasite line. Following addition of rapalog to ∼4–8 hpi ring-stage parasites, *Pf*GCN5::FKBP::GFP was mislocalized from the nucleus to the cytoplasmic face of the parasite plasma membrane in trophozoites and schizonts (Fig. 1D). Growth was significantly inhibited across a 7-day time course (Kruskal–Wallis test, *P* = 0.0023), with a difference in the ratio of normalized parasitemia of mislocalized PfGCN5/control parasites detected from day 6 (Dunn’s multiple comparisons, everything compared with day 0, *P* = 0.0093) (Fig. 1E). Growth inhibition was not due to indirect effects because rapalog had no effect on the growth of WT 3D7 parasites (Supplementary Fig. S2C). From day 3 post-treatment onwards, the proportion of young parasites remained greater than other stages in rapalog treated parasites, whereas vehicle-treated controls cycled normally between early and mid/late parasite stages, suggesting a proportion of the knock sideways parasites were failing to develop from ring stages through to schizonts (Fig. 1F). This was also evident when looking at Giemsa smears, which showed that the parasites did not progress efficiently to the schizont stage, although viable parasites remained detectable until day 6 (Supplementary Fig. S2D).

To assess the importance of the *Pf*GCN5 BRD for growth NF54::diCre-(Pfgcn5:loxP) parasites were synchronized by two incubations with 5% D-sorbitol separated by 12 and 46 h later they were treated with rapamycin. By 26 h post-treatment, the BRD was completely excised (Supplementary Fig. S3A–D). The resulting *Pfgcn5*:ΔBRD parasites had greatly reduced levels of *Pf*GCN5::HA within 24 h and it was undetectable by 72 h post-treatment (Fig. 2B). *Pfgcn5*:ΔBRD growth compared with DMSO-treated controls was significantly reduced over the 112-h cytometry assay (Kruskal–Wallis test, *P* = 0.0017) with the decline significant by 88 h (all timepoints in 112-h assay compared with 16 h, Dunn’s multiple comparison test, *P* = 0.0062) (Fig. 2C). Growth continued to be monitored by microscopy and the ratio declined to negligible levels by 384 h (Fig. 2C) but some *Pfgcn5*:ΔBRD parasites persisted for 20 generations (960 h), after which no parasites were detected (Supplementary Fig. S3E). Growth inhibition of *Pfgcn5*:ΔBRD was not an indirect effect of rapamycin because incubating the NF54::DiCre parental parasites with rapamycin did not affect their growth over 120 h (Supplementary Fig. S3F).

### The *Pf*GCN5 BRD is required for normal development from trophozoite to schizont

By microscopy both *Pfgcn5*:ΔBRD and controls proceeded to differentiate from rings to trophozoites to schizonts throughout the second developmental cycle post-induction, but trophozoites persisted through to the third cycle at higher levels in *Pfgcn5*:ΔBRD than controls (two-way, repeat measure ANOVA variation due to treatment, *P* = 0.0316) (Fig. 2D). To more accurately quantitate the periodicity of the lifecycle, we used flow cytometry to gate parasites into three populations by staining of nucleic acid as low, medium, and high fluorescence intensity (FI) (Supplementary Fig. S4A). Loss of the *Pf*GCN5 BRD caused a slight developmental delay resulting in an extended lifecycle as estimated by the interval between consecutive maximum values of the proportion of parasites that were gated as low FI and interpolated from a cubic spline curve fitted to the 112 h cytometry assay data (Supplementary Fig. S4B). The NF54::diCre-(Pfgcn5:loxP) DMSO-treated parasites had a 48.8-h lifecycle and the *Pfgcn5*:ΔBRD had a 53.7-h lifecycle, consistent with a previous report of a stable *Pfgcn5* BRD deletion parasite line [16].

To quantitate stage specific inhibition of parasite growth, we compared the proportions of the total parasitemia contributed by each of the three FI gated populations during the second lifecycle post-induction i.e. from just after the first invasion (48 h post-induction of knockout) to just before the second invasion (76 h post-induction of knockout) (Fig. 2E). The low FI population was gated on synchronous cultures of early ring stages, but these included some doubly infected RBCs with higher FI. Therefore, this gated low-FI population included lower FI trophozoites at timepoints when very few ring-stage parasites were detectable by microscopy, e.g. 72 and 76 h post-induction (Fig. 2D and E). The *Pfgcn5*:ΔBRD parasites differed from controls in the proportions of high-FI-infected erythrocytes (schizonts) (two-way, repeat measure ANOVA variation due to treatment, *P* = 0.0007) and low-FI-infected erythrocytes (two-way, repeat measure ANOVA variation due to treatment, *P* = 0.004) but there was no difference in the proportions of mid-FI-infected erythrocytes (mature trophozoites) (Fig. 2E). By *post-hoc* Sidak’s multiple comparisons test, the *Pfgcn5*:ΔBRD parasites differed from controls in proportions of high-FI-infected erythrocytes at 72 and 76 h post-induction (*P* = 0.0021 and *P* = 0.0104, respectively) and in proportions of low-FI-infected erythrocytes at 64, 68, 72, and 76 h post-induction (all *P* < 0.0075). From microscopy, the 72 and 76 hpi low-FI-infected erythrocytes were at trophozoite stages. We concluded that lack of the *Pf*GCN5 BRD inhibited progression from trophozoite to schizont stage and some of these trophozoites persisted through to the third cycle. This observation was consistent with the knock-sideways experiment in which the proportion of early stages also increased compared with controls by day 1 post-treatment (Sidak’s *post-hoc* test following two-way repeated measure ANOVA, *P* < 0.0001) (Fig. 1F and Supplementary Fig. S2D). Overall, we concluded that loss of the *Pf*GCN5 BRD caused an acute growth inhibition that resulted in death and was characterized by slowed progression through the cell cycle, consistent with inhibition being primarily due to failure to develop from trophozoite to schizont.

### *Pf*GCN5 inhibits sexual commitment

**Figure 3.**
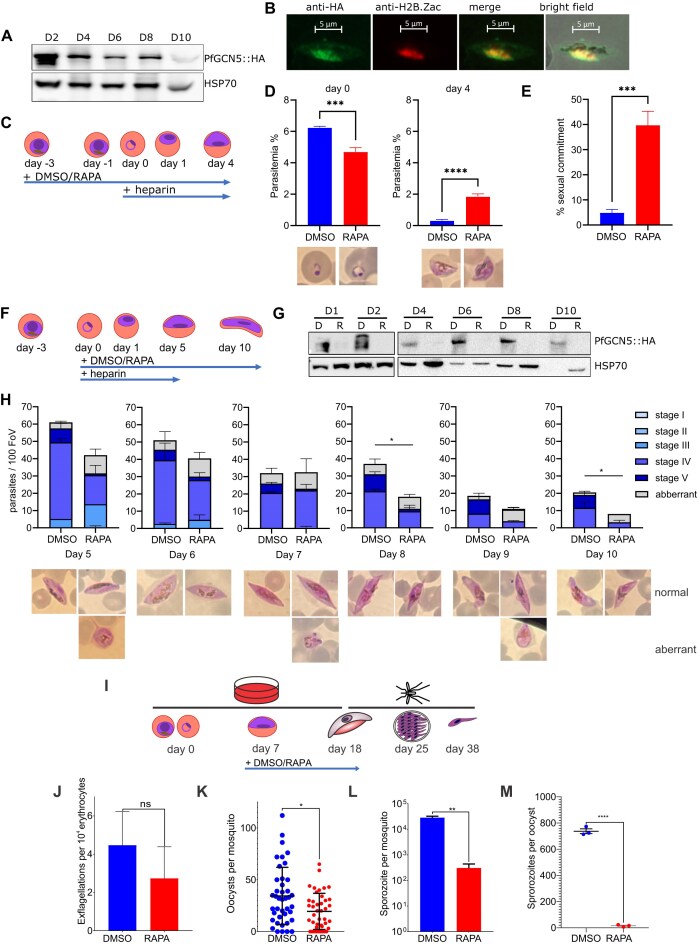
*Pf*GCN5 represses sexual commitment and is also critical for gametocyte maturation and mosquito infection. (**A**) Immunoblot of NF54::diCre-(Pfgcn5:loxP) parasites from day 2–10 of gametocyte differentiation probed with anti-HA and anti-HSP70 as a loading control. (**B**) Immunofluorescence of (stage IV) gametocytes, *Pf*GCN5::HA (green) and H2B.Zac (red). (**C**) Commitment assay: DMSO or RAPA were added to trophozoite stage cultures starting on day –3 and sexual commitment was induced by starvation. After reinvasion on day 0, heparin was added to prevent further asexual replication. (**D**) Conditional deletion of the PfGCN5 BRD in the cycle before commitment results in decreased ring-stage parasitemia at day 0 but increased gametocyte parasitemia at day 4. Representative Giemsa images are shown below. (**E**) Sexual commitment rate calculated based on the parasitemias determined on day 0 (ring) and day 4 (gametocyte). (**F**) Gametocyte maturation assay: sexual conversion was induced by starvation from day –3 onwards and heparin and either RAPA or DMSO were added to synchronous ring-stage cultures on day 0. Gametocyte maturation was monitored until day 10. (**G**) Immunoblot of NF54::diCre-(Pfgcn5:loxP) gametocytes treated with RAPA (R) or DMSO (**D**) from day 0 of gametocytogenesis probed with anti-HA. (**H**) Proportions of gametocyte stages in Giemsa smears from DMSO and RAPA treated cultures from day 5 to day 10 of gametocyte maturation (*n* = 3, unpaired *t*-test). FoV: field of view. Examples of observed normal (upper panel) and aberrant (lower panel) morphologies have been included. (**I**) The *Pf*GCN5 BRD was deleted with rapamycin (RAPA) at day 7 of gametocyte development and controls were treated with DMSO, mosquitoes were fed on the gametocytes at day 18 and oocysts and salivary gland sporozoites counted at days 25 and 38, respectively. (**J**) Male gamete exflagellation (*n* = three experiments, *P* > 0.05, unpaired t-test). (**K**) Oocysts in the mosquito midgut at day 7 after blood meal (oocysts numbers pooled from three experiments, *P* = 0.0121, Mann–Whitney *U*-test). (**L**) Total salivary gland sporozoites (results of three independent trials with sporozoites pooled from 15 mosquitoes, *P* = 0.0021, unpaired *t*-test). (**M**) Salivary gland sporozoites per oocyst (results of three independent experiments with sporozoites pooled from 15 mosquitoes and oocysts measured from 10 mosquitoes, *P* < 0.0001, unpaired *t*-test).

*Pf*GCN5 is present in the parasite nucleus throughout gametocyte development (Fig. 3A and B). To investigate whether *Pf*GCN5 is required for gametocytogenesis we induced deletion of the *Pfgcn5* BRD in highly synchronized NF54::diCre-(Pfgcn5:loxP) trophozoites at day −3, and commitment to gametocytogenesis was induced by starvation until day −1 [32] (Fig. 3C). Heparin was added on day 0 to block asexual parasite invasion and by day 4 the gametocytemia was significantly higher in the *Pfgcn5*:ΔBRD parasites than the DMSO controls (Fig. 3D), consistent with a higher sexual commitment rate of 39.7% in *Pfgcn5*:ΔBRD parasites compared with 5.1% in controls (Fig. 3E). Hence, deletion of the BRD of *Pfgcn5* resulted in increased gametocyte production, consistent with a previous report in which the BRD of *Pfgcn5* was stably deleted [16]. However, through the use of an inducible knockout we have shown that the effect is rapid and dependent on acute loss of the *Pfgcn5* BRD.

### *Pf*GCN5 is required for gametocyte development

Gametocyte development post-commitment requires accurate histone acetylation [28, 57]. To investigate the role of *Pf*GCN5 in gametocyte development, we initiated gametocyte cultures on day –3 and induced knockout of the *Pfgcn5* BRD at day 0 of gametocytogenesis, when the parasites are in the ring stage (Fig. 3F). *Pf*GCN5 was undetectable by day 1 (Fig. 3G) and by day 8 there were significantly fewer stage IV gametocytes in *Pfgcn5*:ΔBRD than in the control (Fig. 3H). By day 10 most gametocytes in the *Pfgcn5*:ΔBRD parasites had aberrant morphology, presenting with round, drop-like or condensed shapes, and no mature stage V gametocytes were detected (Fig. 3H), whereas rapamycin treated NF54::DiCre parental parasites developed normally (Supplementary Fig. S4C). We concluded that *Pf*GCN5 was essential for normal development of stage V gametocytes.

### *Pf*GCN5 is essential for mosquito stage development

To analyse mosquito stage development, *Pfgcn5* BRD deletion was induced with rapamycin at day 7 post-gametocyte induction in mosquito competent gametocyte cultures [31] (Fig. 3I and Supplementary Fig. S4D) and fed to mosquitoes on day 18. The *Pfgcn5*:ΔBRD gametocytes were functional, as measured on day 18 *in vitro* by male exflagellation rates (Fig. 3J), and productively infected mosquitos, as measured by oocyst numbers in dissected mosquito midguts seven days after infection (Fig. 3K). By 20–21 days after infection *Pfgcn5*:ΔBRD parasites produced ∼100x fewer salivary gland sporozoites per mosquito (Fig. 3L) and significantly fewer sporozoites per oocyst (Fig. 3M) than DMSO treated, NF54::diCre-(Pfgcn5:loxP) controls. Rapamycin treatment caused a similar, modest reduction in oocyst numbers in both *Pfgcn5*:ΔBRD parasites (Fig. 3K) and the NF54::DiCre parental line [29] (Supplementary Fig. S4E) but off-target effects of rapamycin treatment did not affect the number of sporozoites produced per mosquito by NF54::DiCre parasites (Supplementary Fig. S4F). We concluded that the loss of the *Pf*GCN5 BRD significantly impacted sporogony.

### The *Pf*GCN5 BRD is required for proper expression of some euchromatic genes

To identify genes regulated by *Pf*GCN5 we compared *Pfgcn5*:ΔBRD to DMSO treated control parasites at 10, 26, 30 and 38 h post-invasion (hpi) during the second cycle after *Pfgcn5* BRD deletion (Fig. 2C) by differential gene expression analysis (DGE) of triplicate RNAseq (Supplementary Tables S1-S3). Average expression profiles of genesets over time also included duplicate samples collected at 34 hpi (Supplementary Table S4). Until 26 hpi samples clustered and correlated best by timepoint, regardless of treatment (Supplementary Fig. S5A and B), after which the *Pfgcn5*:ΔBRD parasites transcriptomes revealed a developmental delay of less than 4 h, consistent with cytometry data (Supplementary Fig. S4B). To address this delay we used RUV [51] to estimate two factors of unwanted variation across the timecourse in two separate analyses. For the first both 26 hpi and 30 hpi DMSO treated parasites were used as controls for the 30 hpi *Pfgcn5*:ΔBRD, and for the second both 34 hpi and 38 hpi DMSO treated parasites were used as controls for the 38 hpi *Pfgcn5*:ΔBRD. This approach removed the variation due to time between the 30 hpi or 38 hpi *Pfgcn5*:ΔBRD and their DMSO treated controls (Supplementary Fig. S6B, C). These factors were then separately included in generalized linear models for DESeq2 differential gene expression analysis at 30 hpi (Supplementary Table S2) and 38 hpi (Supplementary Table S3). There was no delay at 10 and 26 hpi (Supplementary Fig. S6A) so we did not include RUV factors of unwanted variation in these differential gene expression analyses (Supplementary Table S1). No genes were differentially expressed at 10 hpi using a DESeq2-adjusted *P*-value < 0.05 as a significance threshold (data not shown).

**Figure 4.**
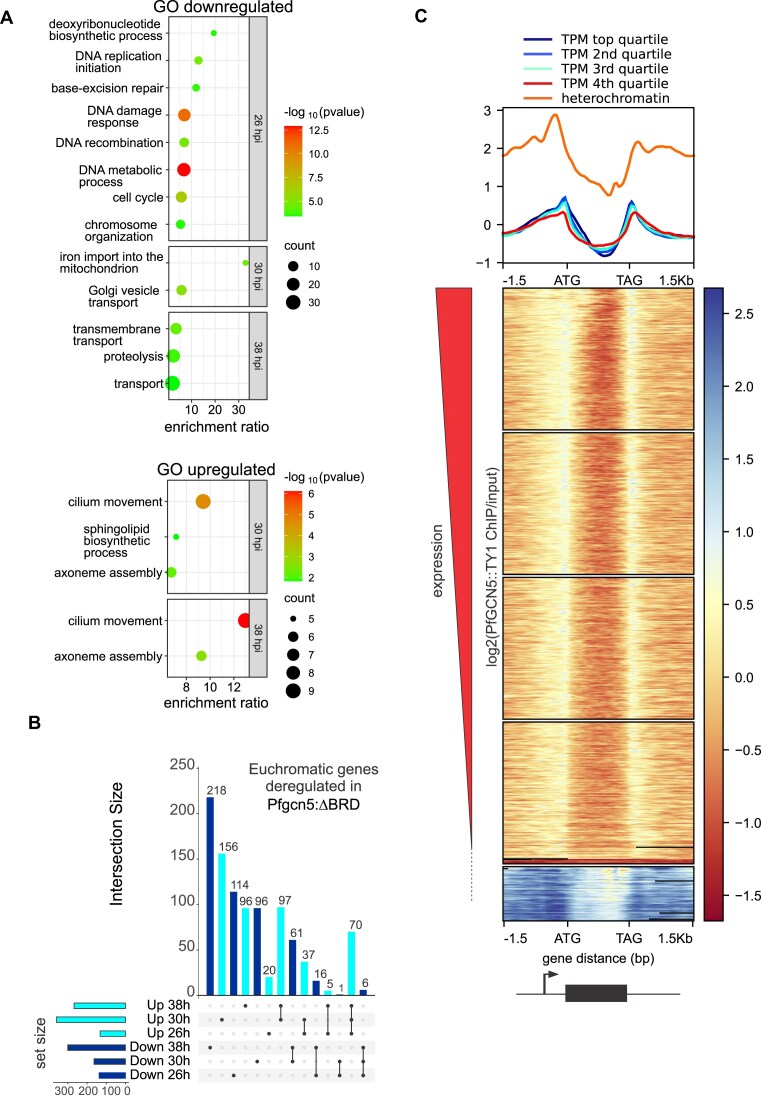
*Pf*GCN5 is enriched in intergenic regions and is most abundant in silent heterochromatin but also regulates expression of euchromatic genes. (**A**) Bubble plots showing the most significant Gene Ontology terms associated with up- or downregulated genes. Each bubble corresponds to a specific GO term and its size represents the count of genes that were differentially expressed. (**B**) UpSet plot showing intersections between deregulated euchromatic genesets at different timepoints in *Pfgcn5*:ΔBRD parasites. (**C**) *Pf*GCN5 enrichment across all genes ranked by descending order of expression and separated into the top four panels by quartile of expression and the bottom panel as silent heterochromatin. Plotted values are mean Bowtie 2 default primary mapped reads of *Pf*GCN5::TY1 log_2_(ChIP/input) (*n* = 2). The mean for each panel of the heatmap is plotted above the heatmap as a line plot.

The 5611 *P. falciparum* genes were segregated into 496 heterochromatic genes and 5115 euchromatic genes by intersection with a published set of H3K9me3 ChIPseq peaks [28]. Across any of the four timepoints used for DGE, 480 euchromatic genes were upregulated and 510 were downregulated (Benjamini–Hochberg FDR < 0.05) (Supplementary Tables S5 and S6). Multiple annotated genesets were deregulated during the asexual lifecycle (Fig. 4A and Supplementary Tables S7–S8) [58]. The most significantly downregulated genesets in 26 hpi parasites related to DNA replication (DNA metabolic process, cell cycle, replication, recombination, and repair) including origin recognition complex subunits, replication factors, DNA polymerase subunits, helicases, condensins, endonucleases, and DNA mismatch repair protein genes. At 30 hpi, genes related to mitochdondrial import of iron were downregulated, including mitoferrin, which is predicted to be essential for the central role of the mitochondria in heme production and metabolism. Ten SNAREs, GTPases and vesicle coat proteins involved in golgi vesicle transport were also downregulated at 30 hpi. The downregulation of fundamental processes of DNA replication, mitochondrial metabolism, and golgi transport in trophozoites was consistent with the inhibited development into schizonts observed in the cell cycle of *Pfgcn5*:ΔBRD. At 38 hpi additional transmembrane transporters were downregulated including multiple mitochondrial transporters of triose-phosphate, NAD, an ABC transporter, and a TIM. In total, 23 genes in the proteolysis pathway were also down-regulated including proteins involved in the proteasome and ubiquitination. This is consistent with the role of PfGCN5 in the stress response [17–19], as also reported in other organisms, and is also consistent with the observed phenotype of *Pfgcn5*:ΔBRD gradual death.

Most downregulated euchromatic genes (428/512) were only downregulated at one timepoint, consistent with PfGCN5 playing a direct role at the promoter of these genes contributing to their activation. However the majority of the upregulated euchromatic genes (272/481) were upregulated at multiple timepoints (Fig. 4B), this could indicate a role for PfGCN5 in indirectly repressing the genes through creating repressive chromatin structures and was reflected in the GO analysis which identified only two upregulated principal pathways in *Pfgcn5*:ΔBRD, and these were shared between 30 and 38 hpi parasites (Fig. 4A, Supplementary Table S8). One pathway was related to sphingomyelin synthesis, indicating the parasites were increasing membrane synthesis, the second was a suite of pathways related to cilium and axoneme production that included 9 dynein and microtubule motor proteins that could be implicated in intracellular transport or cilia production for male gametes, although we note that gametocyte exflagellation was unaffected in *Pfgcn5*:ΔBRD (Fig. 3J).

### *Pf*GCN5 is enriched at gene promoters and is most enriched in heterochromatin

To map *Pf*GCN5 binding sites genome-wide we performed native ChIPseq of *Pf*GCN5::TY1 using 38 h post-invasion, schizont stage 3D7-(Pfgcn5:TY1) parasite nuclei digested by MNase. To correlate the ChIP data with gene expression, we also collected matched RNA samples for RNAseq. From duplicate ChIPs we identified 1906 shared *Pf*GCN5 peaks. *Pf*GCN5 was enriched in intergenic sequence, notably at the start and end of genes (Fig. 4C, Supplementary Fig. S7A, and Supplementary Table S9). In euchromatin *Pf*GCN5 was marginally more enriched at the start and end of genes ranked in the top 75% by expression in the matched RNAseq than in the bottom 25% (Fig. 4C). However, *Pf*GCN5 was most enriched in heterochromatin (Fig. 4C and Supplementary Fig. S7A). Two approaches were used to validate that enrichment of *Pf*GCN5::TY in heterochromatin in this native ChIP was not an artefact due to either mismapping of repetitive subtelomeric sequence or lower coverage of input in heterochromatin than in euchromatin [44]. To address mismapping whilst retaining information from the redundant heterochromatic multigene families, we used Bowtie 2 [42] primary alignments and confirmed the heterochromatic enrichment by comparison to stringent filtring for uniquely mapped reads using BWA [43] with the argument -c 1 (Supplementary Fig. S7B and C). To demonstrate that there was equal read depth of heterochromatin and euchromatin in input we used Mosdepth [47] as previously described to extract read depths [44] (details in ‘Materials and methods’ section). Read depths of heterochromatin and euchromatin were not different for input but were different for anti-Ty ChIP (*P* < 0.0001, Mann Whitney *U* test), confirming the heterochromatic enrichment of *Pf*GCN5 (Supplementary Fig. S7D) [44].

### *Pf*GCN5 modifies chromatin at some euchromatic genes increasing their expression

To investigate the association between *Pf*GCN5 and eukaryotic gene expression we segregated euchromatic genes into the 986 genes that were the closest gene to a *Pf*GCN5 peak summit and were downstream of the summit or had the summit of a *Pf*GCN5 peak in the 5′ one third of their coding sequence, because many peaks spread from the 5′UTR across the start codon. We called these genes proximal to and downstream of *Pf*GCN5 and assumed they were the geneset with promoters most likely to be regulated by *Pf*GCN5. We compared these to the 3728 euchromatic genes that were not the closest gene to a PfGCN5 peak summit or with the 613 euchromatic genes that were the closest gene to a *Pf*GCN5 peak summit but were upstream of it or intersected it within their 3′ one third, in which case the genes were called proximal to and upstream of *Pf*GCN5.

**Figure 5.**
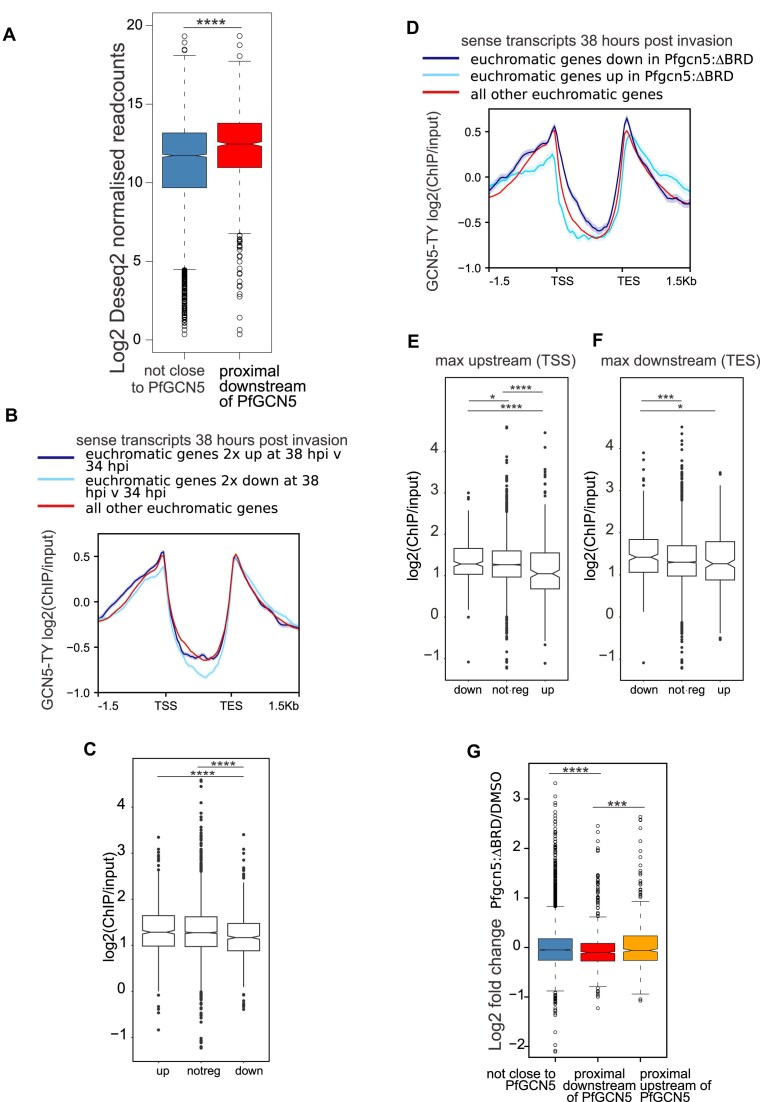
*Pf*GCN5 directly activates euchromatic genes (**A**) Log_2_ DESeq2 size factor normalized readcounts from RNAseq matched samples from *PF*GCN5::TY1 ChIPseq for euchromatic genes proximal to and downstream of a PfGCN5::TY1 peak summit or not close to a PfGCN5::TY1 peak summit. Wilcoxon rank sum test with continuity correction, **** *P* < 0.0001. (**B**) Line plots of mean ± standard error of log_2_(GCN5::TY1 ChIP/input) at 38 hpi from 1.5-kb upstream of the TSS to 1.5-kb downstream of the transcriptional end site (TES) of euchromatic genes that had sense transcripts that were at least two-fold upregulated, or at least two-fold downregulated, or less than two fold deregulated in DMSO treated control NF54::diCre-(Pfgcn5:loxP) at 38 hpi compared with 34 hpi. (**C**) Maximum values of log2 ChIP/input of *Pf*GCN5:TY1 across 50 bp bins of the 1.5 kb 5′ untranslated region of euchromatic genes that were at least 2 fold downregulated (down), or at least twofold upregulated (up) or less than twofold deregulated (notreg) in DMSO-treated control NF54::diCre-(Pfgcn5:loxP) at 38 hpi compared with 34 hpi. Wilcoxon rank sum test with continuity correction, **** *P* < 0.001. (**D**) Line plots of mean ± standard error of log_2_(GCN5::TY1 ChIP/input) at 38 hpi from 1.5-kb upstream to 1.5-kb downstream of euchromatic genes that had upregulated or downregulated or not deregulated sense transcripts in *Pfgcn5*:ΔBRD at 38 hpi. (**E**) Maximum values of log_2_ ChIP/input of *Pf*GCN5:TY1 across 50 bp bins of the 1.5 kb 5′ untranslated region of euchromatic genes that were downregulated (down), or not deregulated (notreg), or upregulated (up) in *Pfgcn5*:ΔBRD. Wilcoxon rank sum test with continuity correction * *P* < 0.05, **** *P* < 0.0001. (**F**) Maximum values of log_2_ ChIP/input of *Pf*GCN5:TY1 across 50 bp bins of the 1.5 kb 3′ untranslated region of euchromatic genes that were downregulated (down), not deregulated (notreg), or upregulated (up) in *Pfgcn5*:ΔBRD. Wilcoxon rank sum test with continuity correction * *P* < 0.05, *** *P* < 0.001. (**G**) Log2 fold change of expression in *Pfgcn5*:ΔBRD compared with DMSO treated controls at 38 hpi of euchromatic genes not close to a summit of PfGCN5::TY1 or proximal and downstream, or upstream, of a summit of PfGCN5::TY1. Wilcoxon rank sum test with continuity correction *** *P* < 0.001, **** *P* < 0.0001.

Consistent with the visual association between euchromatic genes’ expression level and upstream enrichment with *Pf*GCN5 (Fig. 4C), the euchromatic genes proximal to and downstream of *Pf*GCN5 peak summits had higher levels of expression in matched RNAseq of 3D7-(Pfgcn5::TY1) (Supplementary Table S10) than did euchromatic genes that were not closest to, and downstream of, a *Pf*GCN5 peak summit (Wilcoxon rank sum test with continuity correction W = 2184849, *P*-value < 2.2e−16) (Fig. 5A).

To investigate *Pf*GCN5’s regulatory role we integrated the 38 hpi ChIPseq of 3D7-(Pfgcn5::TY1) with RNAseq from the NF54::diCre-(Pfgcn5:loxP) parasites’ timecourse. Firstly we analysed the enrichment of *Pf*GCN5::TY1 at genes that were dynamically regulated across time in the NF54::diCre-(Pfgcn5:loxP) control parasites that expressed PfGCN5. We examined the PfGCN5::TY1 levels at genes that had at least a two-fold change in levels of sense transcripts at 38 hpi (i.e. the same stage as the ChIP samples) relative to 34 hpi in DMSO treated control NF54::diCre-(Pfgcn5:loxP) parasites (Fig. 5B). GCN5 can act both at promoters and at TES so we compared maximum ChIP/input values for 50 bp bins from 1-kb upstream to 1-kb downstream of the transcriptional start sites (TSS) or TES estimated by Stringtie [59] from sense strand RNAseq from matched samples from the ChIPseq. There was less *Pf*GCN5::TY1 at the TSS of the 645 euchromatic genes that were at least two-fold downregulated at 38 hpi relative to 34 hpi compared with the 703 genes that were twofold upregulated or the 3322 genes that were not deregulated (Wilcoxon rank sum test with continuity correction: up versus downregulated W = 194511, *P*-value = 3.228e−06, up versus not regulated W = 932030, *P*-value = 8.3e−08) (Fig. 5B and C). These observations were consistent with the reported activation of gene promoters upon recruitment of *Pf*GCN5 [21, 22] but also demonstrated that *Pf*GCN5 was broadly enriched at euchromatic promoters and was depleted from promoters as transcription declines.

To directly assess the effect of *Pf*GCN5 loss on gene expression we analysed the *Pfgcn5*:ΔBRD parasites. Euchromatic genes that were significantly downregulated were compared with those that were upregulated or not deregulated in *Pfgcn5*:ΔBRD parasites at 38 hpi compared with DMSO controls (Fig. 5D). The maximum levels of log2 ChIP/input *Pf*GCN5::TY1 were marginally higher across the TSS but clearly higher across the TES in euchromatic downregulated genes when compared with euchromatic genes that were not significantly deregulated (Wilcoxon rank sum test with continuity correction TSS: W = 659958, *P*-value = 0.02605. TES: W = 685202, *P*-value = 0.0009305), suggesting *Pf*GCN5 at these sites was directly required for full activation of these genes (Fig. 5E and F). In contrast, genes upregulated in *Pfgcn5*:ΔBRD had lower levels of *Pf*GCN5::TY1 across their TSS and TES than downregulated genes (Wilcoxon rank sum test with continuity correction. TSS: W = 26313, *P*-value = 3.784e−07. TES: W = 31181, *P*-value = 0.01436) or across the TSS but not the TES of all other euchromatic genes (Wilcoxon rank sum test with continuity correction. TSS: W = 409267, *P*-value = 1.978e−07) (Fig. 5E and F), suggesting that *Pf*GCN5 was not directly repressing the promoters of the euchromatic genes that were upregulated in *Pfgcn5*:ΔBRD.

Detecting enrichment of *Pf*GCN5::TY1 at the TSS of genes that were downregulated in *Pfgcn5*:ΔBRD could be hindered by the noise of ChIP experiments. Therefore we analysed the effect of *Pf*GCN5 loss on expression of genes downstream of significant peaks of *Pf*GCN5::TY1 as an orthogonal and statistically robust approach. The log2 fold change of expression at 38 hpi of *Pfgcn5*:ΔBRD/DMSO treated controls was compared between genes that were proximal and downstream or upstream of a *Pf*GCN5::TY1 peak summit, or not closest to a PfGCN5::TY1 peak summit. Deletion of the *Pf*GCN5 BRD resulted in significantly greater downregulation of euchromatic genes that were proximal and downstream of a *Pf*GCN5 binding site than euchromatic genes that were either not proximal to a PfGCN5 binding site (Wilcoxon rank sum test with continuity correction W = 1688074, *P*-value = 1683e−05) (Fig. 5G) or were proximal but upstream of a PfGCN5 binding site (Wilcoxon rank sum test with continuity correction W = 270852, *P*-value = 0.0003241) (Fig. 5G).

The greater downregulation in *Pfgcn5*:ΔBRD of euchromatic genes that were proximal to and downstream of *Pf*GCN5::TY1 peaks compared with other euchromatic genes (Fig. 5G), combined with the slightly greater enrichment of *Pf*GCN5::TY1 at the TSS of genes that were downregulated than genes not deregulated in *Pfgcn5*:ΔBRD (Fig. 5E), indicates that *Pf*GCN5 at the promoter is required for full expression of these euchromatic genes. The clear difference in *Pf*GCN5 levels at 38 hpi between genes expressed at 34 and 38 hpi suggests *Pf*GCN5 is dynamically depleted as the promoter activity wanes (Fig. 5C). Overall, these data are consistent with the significant enrichment of *Pf*GCN5 at euchromatic promoters, which it contributes to activating, and its depletion from promoters as they are inactivated. The conflicting results for 3′ enrichment of *Pf*GCN5 and gene regulation in *Pfgcn5*:ΔBRD prevent inference of a regulatory role of *Pf*GCN5 at the TES, although it is known to regulate plant genes near their TES through modulating H3 acetylation [60].

### Islands of *Pf*GCN5 contain and regulate genes critical for multiple developmental stages

**Figure 6.**
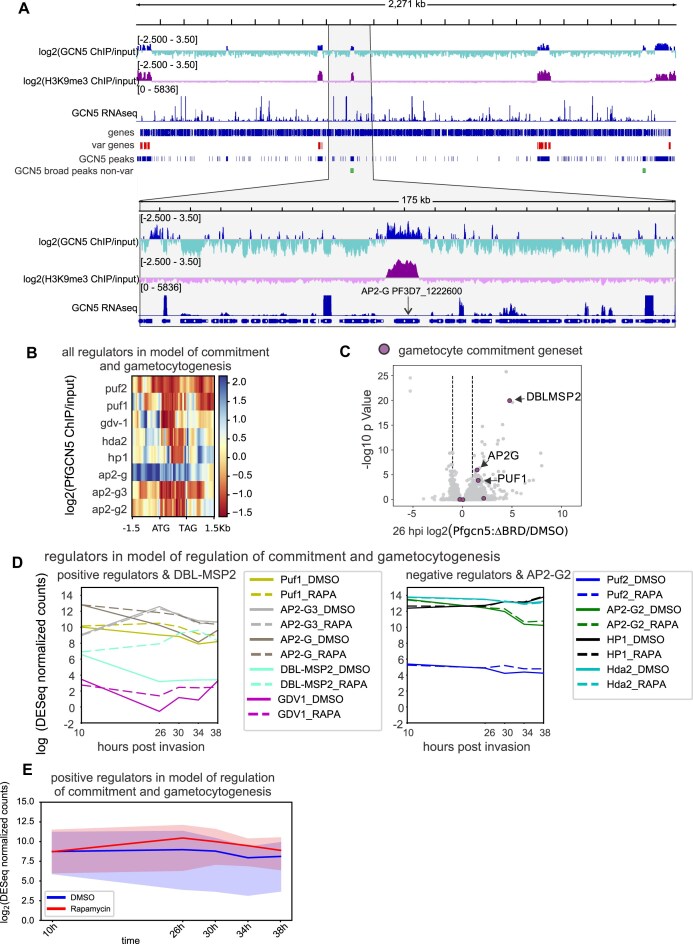
*Pf*GCN5 is enriched in broad peaks at loci that regulate developmental transitions. (**A**) Chromosome 12 with log_2_(ChIP/input) of *Pf*GCN5 and H3K9me3 [28], matched RNAseq from the *Pf*GCN5::TY1 ChIPseq, the positions of all genes, the positions of *var* genes, the positions of consensus peaks of *Pf*GCN5::TY1 enrichment, and the positions of broad *Pf*GCN5::TY1 peaks that are outside *var* gene clusters. The lower panel is a magnification of a broad peak of *Pf*GCN5::TY1 at the *ap2-g* gene. (**B**) Log_2_*Pf*GCN5::TY1 ChIP/input from 1.5-kb upstream to 1.5-kb downstream of the set of regulators in the model of commitment and gametocytogenesis (Malaria Parasite Metabolic Pathways https://mpmp.huji.ac.il/.). (**C**) Differential gene expression (*Pfgcn5*:ΔBRD/DMSO-treated controls) at 26 hpi for all genes with the regulators in the model of commitment and gametocytogenesis and *dblmsp2* [63] indicated. (**D**) Expression [log_2_(DESeq2 normalized readcounts)] over time in hours post-invasion in the second lifecycle following deletion of the *Pf*GCN5 BRD (RAPA) or in controls (DMSO) of the set of regulators in the model of commitment, and gametocytogenesis separated into positive regulators plus *dblmsp2*, and negative regulators plus *ap2-g2*. (**E**) Expression of the positive regulators in the model of commitment and gametocytogenesis (median and interquartile range (IQR ) of log_2_ normalized DESeq2 readcounts in hours post-invasion over the second lifecycle following deletion of the *Pf*GCN5 BRD (RAPA) or in controls (DMSO).

*Pf*GCN5 was strikingly enriched at several, obvious, broad regions that spanned or flanked 25 genes, 14 of which were heterochromatic (Fig. 6A and Supplementary Table S11). The 11 other genes were euchromatic in several H3K9me3 ChIPseq studies that reported ChIPseq peaks [28, 61, 62] but were relatively close to heterochromatin boundaries (seven less than 80 kb, two less than 150 kb from heterochromatin boundaries, Supplementary Table S11), suggesting that these 11 euchromatic islands of *Pf*GCN5 could be involved in maintaining genomic chromatin architecture.

The 25 genes included 14 genes that played important functional roles in stages other than the asexual blood stages and 9 of these were heterochromatic (Supplementary Table S11). The 25 genes that overlapped or were flanked by the *Pf*GCN5 broad regions also included 13 genes that fell within the set of 1238 genes that were deregulated at any timepoint in *Pfgcn5*:ΔBRD (DESeq2-adjusted *P* < 0.05). To test whether the 25 genes associated with PfGCN5 broad regions were overrepresented in the deregulated geneset we analysed 100 000 randomly sampled sets of 25 genes which resulted in an average number of 5.51 genes falling within the deregulated set with an SD of 2.08 (Supplementary Fig. S8A). The calculated proportion of occurrences with at least 13 genes falling in the deregulated set for these 100 000 randomly sampled sets was 0.00101. Thus, genes overlapped or flanked by *Pf*GCN5 broad regions were deregulated in *Pfgcn5*:ΔBRD more than would be expected by chance. Interestingly, several of these genes have been characterized as playing important roles in stage transitions, including the heterochromatic genes *ap2-g* involved in gametocytogenesis and *oocyst rupture protein 1, crmp3, crmp4* involved in oocyst and sporozoite development (Supplementary Table S11). Deregulation of these key genes involved in development might explain the *Pfgcn5*:ΔBRD phenotypes of increased gametocytogenesis, failure to develop mature gametocytes and failure to form sporozoites.

### *Pf*GCN5 represses gametocytogenesis regulators

The known gametocytogenesis inducers *ap2-g* and *gdv-1* were enriched in *Pf*GCN5, as was the gametocyte protein *puf1* (Fig. 6A and B and Supplementary Fig. S8B)*. ap2-g* and *dblmsp2*, which was also recently shown to be activated by GDV1 [63], were amongst the genes associated with broad regions of *Pf*GCN5 and were also amongst the most significantly upregulated genes in *Pfgcn5*:ΔBRD (Fig. 6C, Supplementary Fig. S8C, and Supplementary Table S10). Deregulation of *ap2-g, dblmsp2*, and *gdv1* in *Pfgcn5*:ΔBRD may have been an indirect consequence of disrupted heterochromatin at these loci. Most of the core set of genes with proven roles in direct, positive regulation of gametocytogenesis in *P. falciparum* (Supplementary Table S12) [34] were upregulated in *Pfgcn5*:ΔBRD (Fig. 6D and E) but negative regulators were not (Fig. 6D), consistent with the increased sexual commitment in *Pfgcn5*:ΔBRD (Fig. 3). *ap2-g3* expression was unaffected in the *Pfgcn5*:ΔBRD parasites but its role in gametocytogenesis is contentious.

### The *Pf*GCN5 BRD is required for proper repression of heterochromatic genes

**Figure 7.**
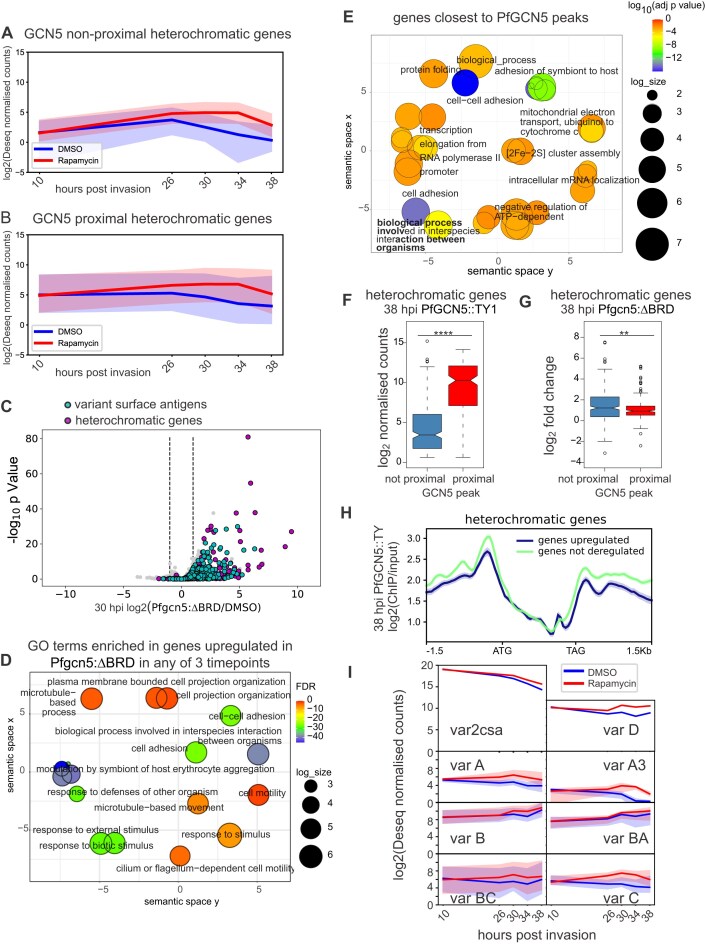
Heterochromatic genes are partially derepressed in *Pfgcn5*:ΔBRD regardless of proximity to *Pf*GCN5. RNAseq of *Pfgcn5*:ΔBRD and of DMSO-treated controls at different times post-induction plotted for heterochromatic genes that (**A**) were not, or (**B**) were the closest gene to a peak of *Pf*GCN5. Median and IQR of log_2_(normalized DESeq2 readcounts). (**C**) Differential gene expression (DESeq2) of *Pfgcn5*:ΔBRD parasites compared with controls at 30 hpi with heterochromatic variant surface antigen genes, and other heterochromatic genes, separately highlighted. (**D**) REVIGO plot summarising the pathway terms enriched in genes upregulated across any of the three timepoints used for differential gene expression. (**E**) REVIGO plot summarizing pathway terms enriched in genes closest to, and within 2 kb of, a *Pf*GCN5 peak. For both, (panels D and E)), Multidimensional Scaling (MDS) was used to reduce the dimensionality of a matrix of the GO terms pairwise semantic similarities [54], circle size reflects size of the background set of the GO term; log_10_(adjusted p value) from Fishers exact test. (**F**) Expression levels (log_2_ normalized readcounts (DESeq2)) of heterochromatic genes in 38 hpi *Pf*GCN5::TY1 parasites that were the closest (proximal) gene, or not, to a peak of *Pf*GCN5::TY1 (****P < 0.0001 Mann–Whitney *U*-test). (**G**) Log_2_ fold change in expression of heterochromatic genes that were the closest (proximal) gene, or not, to a peak of *Pf*GCN5 in *Pfgcn5*:ΔBRD parasites relative to DMSO treated controls at 38 hpi (**P < 0.01 Mann Whitney *U*-test). (**H**) Mean ± SE log_2_(GCN5::TY1 ChIP/input) at 38 hpi from 1.5-kb upstream to 1.5-kb downstream of heterochromatic genes that were at least twofold upregulated and all other not deregulated heterochromatic genes in *Pfgcn5*:ΔBRD at 38 hpi. (**I**) Expression (DESeq2 normalized readcounts) of single *var* genes and median and IQR for *var* multi-gene groups [76] during the second lifecycle following deletion of the *Pf*GCN5 BRD in *Pfgcn5*:ΔBRD (rapamycin) or controls (DMSO).

Heterochromatic genes were de-repressed in trophozoite and schizont stage *Pfgcn5*:ΔBRD parasites regardless of proximity to *Pf*GCN5 (Fig. 7A and B). Unsurprisingly, only two heterochromatic genes were downregulated but 244 were upregulated and nearly all of these were upregulated at multiple timepoints (Supplementary Fig. S8D and Supplementary Table S13). Combining the three timepoints used for differential expression (26, 30 and 38 hpi) a significantly greater proportion of heterochromatic than euchromatic genes were upregulated in the *Pfgcn5*:ΔBRD parasites (244/496 and 481/5113 upregulated respectively, Fisher’s exact test, *P* < 0.0001).

The heterochromatic, variant surface antigen multigene families *var, rif* and *stevor* and the *pfmc2tm* genes were de-repressed in trophozoite and schizont stage *Pfgcn5*:ΔBRD (Fig. 7C) and were the most significant GO terms (modulation by symbiont of host erythrocyte aggregation) enriched in all de-repressed genes (Supplementary Table S14, Fig. 7D). Pathways related to heterochromatic gene families (adhesion of symbiont to host, cell adhesion, cell-cell adhesion) were also the most significantly over-represented GOs amongst all genes closest to *Pf*GCN5 peaks (Fig. 7E), and a significantly greater proportion of heterochromatic (237/496) than euchromatic (1394/5115) genes were closest to a *Pf*GCN5 peak summit (Fishers’ exact test, *P* < 0.0001).

The above findings suggested *Pf*GCN5 repressed heterochromatic genes, but within heterochromatin, proximity to *Pf*GCN5 peaks was associated with higher levels of gene expression in matched RNAseq from 3D7-(Pfgcn5::TY1) parasites (log_2_Deseq2 size factor normalized readcounts, Wilcoxon rank sum test with continuity correction W = 41 703, *P*-value < 2.2e−16) (Fig. 7F) and less gene de-repression in *Pfgcn5*:ΔBRD/DMSO control (fold change, Wilcoxon rank sum test with continuity correction W = 26 038, *P*-value = 0.009 667) (Fig. 7G). The heterochromatic genes that were at least two fold upregulated in *Pfgcn5*:ΔBRD parasites were also less enriched in *Pf*GCN5 at their promoters than other heterochromatic genes in 3D7-(Pfgcn5::TY1) (Fig. 7H). These data all suggested that *Pf*GCN5 was not directly repressing genes in heterochromatin.

### *Pf*GCN5 exerts low-level repression of the heterochromatic *var* multi-gene family but is not required for their proper regulation

To investigate whether *Pf*GCN5 affected the specific, coordinated regulation of heterochromatic genes, we analysed the *var* multi-gene family. Full length transcripts of a single *var* gene are expressed in ring-stage parasites [64, 65]. At 10 hpi ring stage, both *Pfgcn5*:ΔBRD and DMSO treated control parasites expressed approximately 500-fold more (Fig. 7I) of full-length (Supplementary Fig. S9) *var2csa* than any other *var* gene, indicating that presence of the *Pf*GCN5 BRD in ring stages was unnecessary for proper *var* expression. From ∼20 hpi, WT trophozoites no longer express full length, functional *var* transcripts but instead transcribe sterile transcripts of the second exon from multiple *var* genes [66, 67]. By 38 hpi many *var* genes were slightly upregulated in *Pfgcn5*:ΔBRD parasites (Fig. 7I), but, aside from full-length *var2csa*, these were the same sterile, second exon transcripts that the controls also expressed (Supplementary Fig. S9). The relaxation of second exon repression, but maintenance of allelic exclusive expression of full length *var* genes, is consistent with partially disrupted heterochromatin structure.

### *Pf*GCN5 is required for proper acetylation of the alternative histone Pf H2B.Z and its distribution in chromatin

**Figure 8.**
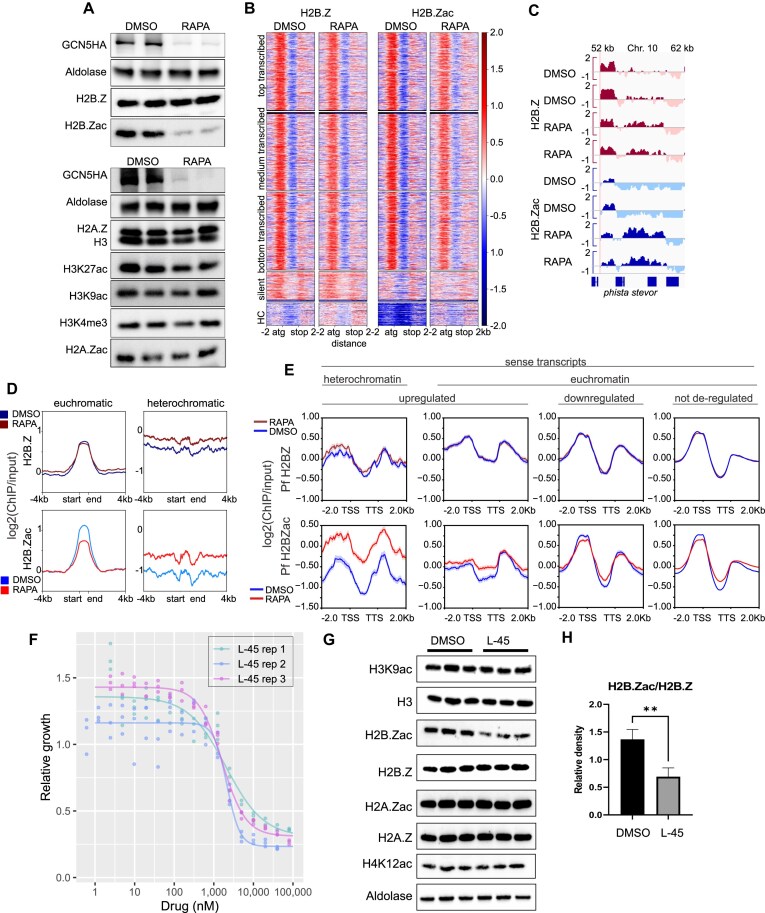
*Pf*GCN5 regulates Pf H2B.Z acetylation. (**A**) Immunoblot of histone modifications and aldolase in *Pfgcn5*:ΔBRD (RAPA) and controls (DMSO), *n* = 2 biological replicates. (**B**) Pf H2B.Z and Pf H2B.Zac (log_2_ ChIP/Input) in *Pfgcn5*:ΔBRD and controls plotted across all genes and their 2-kb up/downstream regions ranked by gene expression levels in trophozoite-stage parasites and separated by expression tertile or as silent euchromatic or heterochromatic (HC) [28]. (**C**) log_2_ (ChIP/Input) across a region containing a *phista* and a *stevor* gene (*n* = 2). (**D**) Average signal for Pf H2B.Z and Pf H2B.Zac log_2_ (ChIP/Input) over *Pf*GCN5::TY1 peaks in heterochromatic or euchromatic loci. (**E**) Average log_2_-transformed ChIP/Input signals from Pf H2B.Z and Pf H2B.Zac in *Pfgcn5*:ΔBRD and controls across genes up- or downregulated or not deregulated 38 hpi after deletion of the *Pf*GCN5 BRD. (**F**) *Plasmodium falciparum* growth in the presence of the small compound L-45. (**G**) Immunoblot of *Pfgcn5*:ΔBRD and controls grown for 4 h in the presence of L-45 and probed with antibodies to the indicated proteins (*n* = 3). (**H**) Densitometry of Pf H2B.Zac/ Pf H2B.Z ratio from the western blot in panel (H), mean, and SD, unpaired *t*-test **P< 0.01.

Loss of the *Pf*GCN5 BRD could disrupt targeting of histone acetylation causing gene deregulation. *Pf*GCN5 was predicted to acetylate H3K9 [16, 68], and indirectly lead to H3K4me3 [16], so the effect of *Pf*GCN5 BRD deletion on histone acetylations and H3K4me3 was examined at 34–40 hpi in the cycle following induction of deletion of the *Pfgcn5* BRD. Pf H2B.Z acetylation was dramatically reduced (Fig. 8A) but other histone modifications were not clearly affected, suggesting that H3K9, H3K27 and Pf H2A.Z were either not acetylated by *Pf*GCN5 at this time point or that the *Pf*GCN5 BRD was not required for targeting *Pf*GCN5 to these nucleosomes (Fig. 8A). PfH2B.Zac was also decreased and H3K9ac unaffected at 32 hpi in the 3D7-(Pfgcn5::Fkbp::Gfp) parasites after knock sideways of *Pf*GCN5::FKBP::GFP (Supplementary Fig. S10A). The decrease of Pf H2B.Zac in the *Pf*GCN5 knock sideways was more modest than in *Pfgcn5*:ΔBRD, probably because compared with the nearly complete *Pf*GCN5 BRD knockout (Fig. 8A), knock sideways was always incomplete and some functional *Pf*GCN5 persisted in the nucleus (Fig. 1D).

We used the same 34–40 hpi *Pfgcn5*:ΔBRD parasite cultures for cross-linked ChIP of total Pf H2B.Z and acetylated Pf H2B.Z. In DMSO treated control parasites the patterns of euchromatic, 5′ intergenic enrichment and heterochromatic depletion were as predicted [14, 27, 69] (Fig. 8B, Supplementary Tables S15-S18). Compared with controls, the *Pfgcn5*:ΔBRD parasites (RAPA) were relatively depleted of acetylated Pf H2B.Z in euchromatin but enriched in both total and acetylated Pf H2B.Z in heterochromatin (Fig. 8B, Supplementary Fig. S10B), as illustrated at a heterochromatic locus spanning *phista* and *stevor* genes (Fig. 8C).

The re-distribution of total and acetylated Pf H2B.Z in different chromatin compartments after deletion of the *Pf*GCN5 BRD was further examined across *Pf*GCN5::TY1 ChIPseq peaks in *Pfgcn5*:ΔBRD (Fig. 8D). Total and acetylated Pf H2B.Z were both enriched at euchromatic peaks of *Pf*GCN5::TY1 (Fig. 8D) but acetylated Pf H2B.Z levels were lower at these peaks in *Pfgcn5*:ΔBRD than in control parasites, consistent with *Pf*GCN5 playing a direct role in the acetylation of Pf H2B.Z in euchromatin. In contrast, the increased levels of heterochromatic total and acetylated Pf H2B.Z in *Pfgcn5*:ΔBRD (Supplementary Fig. S10B) were not clearly confined to the peaks of *Pf*GCN5::TY1 (Fig. 8D). This indicated that *Pf*GCN5 BRD deletion either indirectly caused the increased levels of total and acetylated Pf H2B.Z in *Pfgcn5*:ΔBRD heterochromatin or it disrupted *Pf*GCN5 targeting and allowed *Pf*GCN5 to dramatically re-localise to heterochromatin in *Pfgcn5*:ΔBRD compared with the *Pf*GCN5::TY1 parasites used for ChIPseq.

Levels of acetylated but not total Pf H2B.Z are associated dynamically with gene expression [14]. Therefore we investigated whether genes that had altered expression at 38 hpi in *Pfgcn5*:ΔBRD parasites had differences in levels of total and acetylated Pf H2B.Z between *Pfgcn5*:ΔBRD and controls. Indeed, Pf H2B.Zac was enriched across the upregulated euchromatic (*n* = 307) and heterochromatic (*n* = 264) genes in *Pfgcn5*:ΔBRD parasites (Fig. 8E). Heterochromatic Pf H2B.Z was also enriched relative to DMSO controls, whereas there was no change in Pf H2B.Z in upregulated euchromatic genes (Fig. 8E). The levels of Pf H2B.Z were unaffected in *Pfgcn5*:ΔBRD parasites at euchromatic genes that were downregulated or not deregulated, but Pf H2B.Zac was depleted around their promoters (Fig. 8E). Consequently, at gene promoters *Pf*GCN5 modulated Pf H2B.Zac levels, which are associated with dynamic gene expression across time [14].

### Treatment with the small compound L-45 phenocopies deletion of the *Pf*GCN5 BRD

The compound L-45 binds the recombinant *Pf*GCN5 BRD [70], so we investigated whether treatment with L-45 would phenocopy the asexual growth inhibition and the reduced acetylation of Pf H2B.Z of *Pfgcn5*:ΔBRD parasites. Treatment with L-45 for 72 h inhibited asexual parasite growth (geometric mean IC_50_= 1908 nM 95% CI 1314–2771 nM) (Fig. 8F) and treatment of 24 hpi trophozoites with L-45 for 4h indeed caused a partial and specific inhibition of Pf H2B.Z acetylation (Fig. 8G,H). This further corroborates Pf H2B.Z as a key target of *Pf*GCN5 mediated acetylation.

## Discussion

We show for the first time that *Pf*GCN5 is required upstream of some euchromatic genes for their proper activation but is also most abundant in heterochromatin and appears to repress heterochromatic genes indirectly through maintaining heterochromatin structure. We also show for the first time that *Pf*GCN5 is dramatically enriched at a handful of broad loci that contain genes important for developmental transitions and that parasites lacking the *Pf*GCN5 BRD are unable to properly regulate these transitions.

### *Pf*GCN5 regulates development

Mislocalisation of *Pf*GCN5 or loss of its BRD both cause a proportion of asexual parasites to fail to develop into schizonts which appears to be the cause of death in these parasites. The *Pf*GCN5 BRD is required for repression of the AP2-G master regulator of gametocytogenesis, but the *Pf*GCN5 BRD is also required for development of gametocytes and development of oocysts to sporozoites. These defects were consistent with striking patterns of *Pf*GCN5 enrichment at genes important for developmental transitions and altered patterns of gene expression in *Pfgcn5*:ΔBRD parasites.

### *Pf*GCN5 at euchromatic gene promoters affects their expression

A proportion of *Pfgcn5*:ΔBRD parasites survived for over one month. Their gradual death is consistent with a role for *Pf*GCN5 in maintenance of epigenetic marks and the gradual loss of partially functionally redundant histone acetylations in *Pfgcn5*:ΔBRD parasites. The disordered N terminal end of *Pf*GCN5 could be involved in protein-protein interactions, so it is possible that truncated *Pf*GCN5 could still be recruited to the SAGA complex in *Pfgcn5*:ΔBRD and that other components of SAGA, e.g. the proposed H3K4me3 binding PHD1 [20], enable the impaired complex to partially fulfill its important role in initiating transcription. PfGCN5 is enriched at euchromatic promoters regardless of dynamic gene expression and only dynamically depleted as the promoter activity wanes. This is consistent with the atypically acetylated state of the non-expressed *P. falciparum* euchromatic genome [9]. It is also consistent with our previous observations that H3 and Pf H2B.Z are acetylated at euchromatic promoters at approximately the start of S phase, often prior to gene expression, and are maintained in an acetylated state until after gene expression has ceased [12, 14]. This could also explain why the comparisons between promoters of euchromatic genes that were downregulated and not deregulated in *Pfgcn5*:ΔBRD revealed only slight variation in PfGCN5 levels in 3D7-(Pfgcn5::TY1) schizonts and similar levels of Pf H2B.Zac depletion in *Pfgcn5*:ΔBRD. This lack of differences would be detected if the majority of the not deregulated gene promoters that were dynamically enriched with PfGCN5 were not yet expressed.

### *Pf*GCN5 is enriched in heterochromatin where it is required for gene silencing

BRDs are typically associated with acetylated histone lysines in euchromatin but there are exceptions, e.g. the *Trypanosoma brucei* BRD proteins Bdf2 and Bdf3 are enriched at repressed, heterochromatic *vsg* genes [71]. De-repression of some heterochromatic genes in parasites many generations after loss of the *Pf*GCN5 BRD has been previously noted, but in the absence of ChIPseq, no conclusion could be drawn as to how *Pf*GCN5 affected heterochromatic gene expression [16]. We have now shown that proximity to *Pf*GCN5 is associated with higher levels of gene expression in heterochromatin and that the heterochromatic genes derepressed in *Pfgcn5*:ΔBRD parasites had lower levels of *Pf*GCN5 at their promoters than other heterochromatic genes in 3D7-(Pfgcn5::TY1) parasites. This suggests that the *Pf*GCN5 complex indirectly represses these genes through maintenance of heterochromatin structure.

### *Pf*GCN5 could be recruited to heterochromatin indirectly and form boundaries

The *Pf*GCN5 containing SAGA-like complex contains a PHD2 domain that might bind H3K9me3 or H3K36me3 in silent heterochromatin [16, 20]. PHD2 and *Pf*GCN5 also bind the SIP2 protein that binds DNA motifs in telomeric heterochromatin [20]. Within the SAGA-like complex, *Pf*GCN5 associates with the Ada2 subunit [20]. In yeast Ada2 also associates with repressive heterochromatin at telomeres in association with SAGA or a related HAT complex and GCN5 and Ada2 antagonise the further spread of silent heterochromatin [26]. These precedents suggest *Pf*GCN5 could function at heterochromatin to maintain chromatin boundaries. Chromatin boundary dynamics appear to be the main regulator of clonally variant gene expression in *P. falciparum* [72].

### Pf H2B.Zac enrichment in *Pfgcn5*:ΔBRD heterochromatin implicates *Pf*GCN5 in boundary function

**Figure 9.**
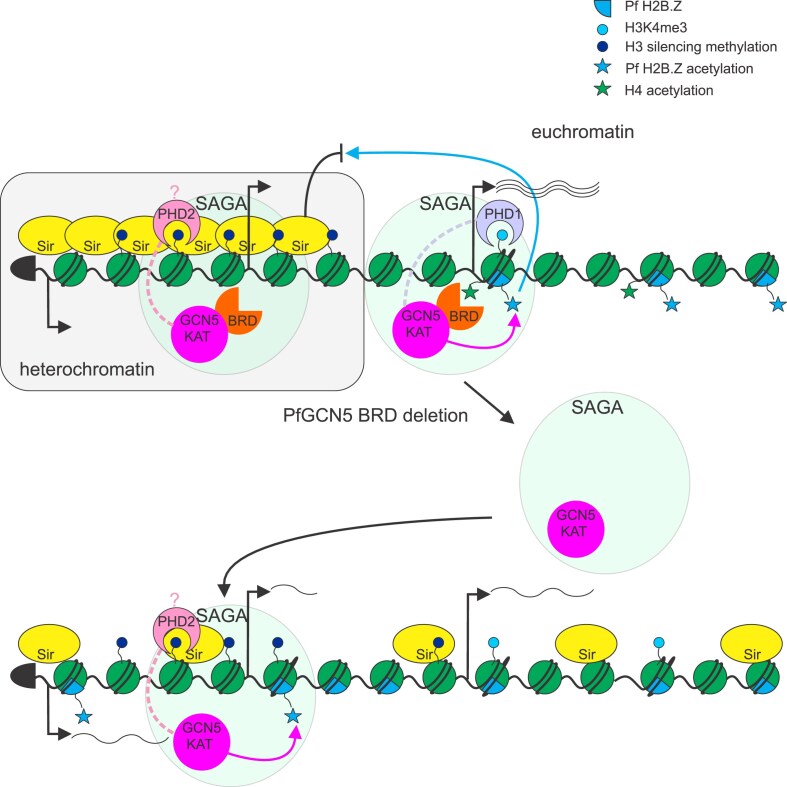
Model of the effect of *Pf*GCN5 BRD deletion on chromatin structure and gene expression. *Pf*GCN5 binds multi-acetylated H4 and PHD1 binds H3K4me3 to recruit the SAGA-like complex to euchromatic promoters. *Pf*GCN5 partially acetylates nucleosomal Pf H2B.Z. The SAGA-like complex and nucleosomes containing Pf H2B.Z form a boundary, preventing the spread of Sir2-dependent heterochromatin. *Pf*GCN5 also binds PHD2 which possibly recruits SAGA to methylated histone lysines in Sir2-dependent heterochromatin. Deletion of the *Pf*GCN5 BRD prevents the SAGA-like complex binding to multi-acetylated H4 at euchromatic promoters, supresses gene expression and reduces Pf H2B.Z acetylation. Deletion of the *Pf*GCN5 BRD could also result in loss of the boundary which allows heterochromatin to be depleted of Sir2 and enriched in Pf H2B.Z. Within the disrupted heterochromatin genes are de-repressed regardless of proximity to *Pf*GCN5. The increased soluble pool of SAGA-like complex might also bind heterochromatin via the PHD domain proteins and acetylate Pf H2B.Z.

In *S. cerevisae* the SWR1 complex binds acetylated H4 and deposits H2A.Z-H2B dimers [24] and H2A.Z is subsequently, partially acetylated by GCN5 [23, 73]. *Pf*GCN5 interacts with PfSwr1 [20] and the *Pf*GCN5 BRD also binds H4K5acK8acK12ac [20]. Loss of targeting of *Pf*GCN5 to acetylated H4 could cause the depletion of Pf H2B.Z acetylation in *Pfgcn5*:ΔBRD euchromatin. This could also cause loss of the SAGA-like complex at the boundary of euchromatin and telomeric heterochromatin. Interestingly, loss of GCN5 [26] or H2A.Z [25] in yeast disrupted this boundary and caused inward spreading of Sir2. The resulting depletion of Sir2 from telomeres was proposed to disrupt silencing of telomeric heterochromatin [26]. A similar loss of this boundary in *Pfgcn5*:ΔBRD could cause depletion of PfSir2A and B from the telomeres and the observed enrichment of acetylated Pf H2B.Z in heterochromatin (Fig. 9). Indeed, we recently showed that loss of PfSir2A or B results in incursion of acetylated Pf H2B.Z into heterochromatin [14]. The unbound pool of *Pf*GCN5 lacking a BRD could also drive a greater association of the SAGA-like complex with heterochromatin via its PHD proteins where it could promote the aberrant incorporation and acetylation of PfH2B.Z in heterochromatin (Fig. 9).

### *Pf*GCN5 is not extensively processed and is not the sole catalyst of acetylation of H3K9 in early schizonts

Cleavage of *Pf*GCN5 has been reported to be important for nuclear transport of *Pf*GCN5 in the 3D7 clone [56]. We also observed cleaved *Pf*GCN5 of approximately 70 and 40 kDa in some samples of the NF54::diCre-(Pfgcn5:loxP), which is a subclone of the NF54 isolate from which 3D7 was cloned (Fig. 2B, 72 and 120 h post-treatment DMSO control). However, full-length *Pf*GCN5 was clearly transported to the nucleus and we did not observe the nearly complete processing previously reported [56] (Fig. 1B and C and Supplementary Fig. S1C). The processing was previously reported using polyclonal antibodies to the C terminal 375 amino acids of *Pf*GCN5 [56], so if processed forms also lacked some portion of the C terminus we would not detect them with our C terminal 3xHA and TY1 epitope tags. Inhibition of *Pf*GCN5 cleavage was also reported to inhibit acetylation of H3K9 [56] but we found that H3K9ac is largely unaffected by induced loss of the *Pf*GCN5 BRD or *Pf*GCN5 knock sideways, suggesting that *Pf*GCN5 is not the sole catalyst of H3K9 acetylation.

### *Pf*GCN5 BRD is essential for most parasites

The inability of the *Pfgcn5*:ΔBRD parasites to survive more than 20 generations without the *Pf*GCN5 BRD differs from a report describing *Pf*GCN5 BRD knockouts that were recovered by FACS [16]. The NF54 isolate was the parent of both the NF54::diCre-(Pfgcn5:loxP) subclone that we used and the 3D7 clone used by Miao *et al.*, so we suspect that they recovered a rare mutant capable of compensating for loss of the *Pf*GCN5 BRD. Redundancy of the *Pf*GCN5 BRD seems plausible because Pf H2B.Z is still acetylated, albeit at decreased levels, in *Pfgcn5*:ΔBRD and the PHD domains could provide the SAGA-like complex with other targeting modules.

The small compound L-45 does not kill human cells [70] but it binds the BRDs of *Pf*GCN5 [70] and *Toxoplasma gondii* GCN5b and kills *T. gondii* [74]. Here, we show that L-45 is moderately potent against *P. falciparum* and phenocopies deletion of the *Pf*GCN5 BRD. The slow demise of asexual blood-stage parasites lacking their BRD suggests *Pf*GCN5 BRD inhibitors may not be ideal drugs, but the inability of *Pfgcn5*:ΔBRD parasites to form infective stage V gametocytes and the inhibition of sporozoite development suggests that *Pf*GCN5 BRD inhibitors could be useful as transmission blocking drugs.

## Supplementary Material

gkaf218_Supplemental_Files

## Data Availability

Sequences are available in the Short Read Archive BioProject: PRJNA1043853.

## Notes

Present address: Cambridge University, Cambridge CB2 1TN, United Kingdom

Present address: ZiP Diagnostics, Collingwood, Victoria 3066, Australia

present address: australian red cross lifeblood, west melbourne, victoria 3003, australia
