# Correction to ‘PfGCN5 is essential for Plasmodium falciparum survival and transmission and regulates Pf H2B.Z acetylation and chromatin structure’

**DOI:** 10.1093/nar/gkaf309

**Published:** 2025-04-23

This is a correction to: Jingyi Tang, Lee M Yeoh, Myriam D Grotz, Christopher D Goodman, Jingyi Tang, Hanh H T Nguyen, Chunhao Yu, Kapil Pareek, Fairley McPherson, Anton Cozijnsen, Samuel A Hustadt, Gabrielle A Josling, Karen P Day, Danae Schulz, Geoffrey I McFadden, Tania F de Koning-Ward, Michaela Petter, Michael F Duffy, *Pf*GCN5 is essential for *Plasmodium falciparum* survival and transmission and regulates Pf H2B.Z acetylation and chromatin structure, *Nucleic Acids Research*, Volume 53, Issue 6, 11 April 2025, gkaf218, https://doi.org/10.1093/nar/gkaf218.

The originally published HTML version of this manuscript contained two errors that were introduced during production:

1. Author Scott A. Chisholm was removed from the author list and author Jingyi Tang was duplicated in Dr Chisholm's place.
2. Figures 1 & 2 were incorrectly placed in the Materials and methods section instead of the Results section.

The publisher apologizes for these errors, which have been corrected.

Additionally, an incorrect affiliation was listed for author Jingyi Tang. This has been removed.

