# Supplementary material for: PfGCN5 is essential for Plasmodium falciparum survival and transmission and regulates Pf H2B.Z acetylation and chromatin structure
Source: Nucleic Acids Res. 2025 Mar 29;53(6):gkaf218. doi: 10.1093/nar/gkaf218 (PMC11954527; doi:10.1093/nar/gkaf218)
Supplement: gkaf218_Supplemental_Files [file gkaf218_supplemental_files.zip › suppl_table_legends_suppl_figs_and_legends_R3.pdf]

Supplementary table 1 Differential gene expression analysis of biological triplicates of (*Pfgcn*5:ΔBRD/DMSO control) at 26 hours post invasion (64 hours post induction of knockout of the *Pfgcn5* bromodomain)

Supplementary table 2 Differential gene expression analysis of biological triplicates of (*Pfgcn*5:ΔBRD/DMSO control) at 30 hours post invasion (68 hours post induction of knockout of the *Pfgcn5* bromodomain)

Supplementary table 3 Differential gene expression analysis of biological triplicates of (*Pfgcn*5:ΔBRD/DMSO control) at 38 hours post invasion (76 hours post induction of knockout of the *Pfgcn5* bromodomain)

Supplementary table 4 Size factor normalised log2 readcounts from Deseq2 of RNAseq of all replicates from the *Pfgcn5*:ΔBRD and DMSO control timecourse, samples from 10, 26, 30, 34 and 38 hpi.

Supplementary table 5 Euchromatic genes upregulated in differential gene expression analysis of biological triplicates of (*Pfgcn5*:ΔBRD/DMSO control) at any of 26, 30 or 38 hours post invasion

Supplementary table 6 Euchromatic genes downregulated in differential gene expression analysis of biological triplicates of (*Pfgcn5*:ΔBRD/DMSO control) at any of 26, 30 or 38 hours post invasion

Supplementary table 7 Gene ontology analysis of euchromatic genes downregulated in *Pfgcn5*:ΔBRD compared to DMSO controls at 26, 30 and 38h post invasion. P values are from Fishers exact tests, FDR from Benjamini Hochberg. Nested terms are redundant.

Supplementary table 8 Gene ontology analysis of euchromatic genes upregulated in *Pfgcn5*:ΔBRD compared to DMSO controls at 26, 30 and 38h post invasion. P values are from Fishers exact tests, FDR from Benjamini Hochberg. Nested terms are redundant.

Supplementary table 9 Genes closest to summits of MSPC consensus peaks of ChIP PfGCN5::TY1/input.

Supplementary table 10 Size factor normalised log2 readcounts from Deseq2 of RNAseq of both replicates from the PfGCN5::TY1 schizont ChIP.

Supplementary table 11 Genes intersecting or near broad GCN5 region.

Supplementary table 12 Genes implicated in gametocytogenesis and gametocyte development. Genes that are proposed to positively contribute to gametocytogenesis or gametocyte development plus the gametocytogenesis repressors HP1 and HDA2 obtained from curated lists (76) of "gametocyte development" (78), "gametocyte IMC and plate formation" (79), "Model for the regulation of commitment and gametocytogenesis" (34), protein export in gametocytes" (80), "the signalling pathway involved in gametocytogenesis"(78).

Supplementary table 13 Heterochromatic genes upregulated in differential gene expression analysis of biological triplicates of (Pfgcn5:ΔBRD/DMSO control) at any of 26, 30 or 38 hours post invasion.

Supplementary table 14 Gene ontology analysis of heterochromatic genes up-regulated in Pfgcn5:ΔBRD compared to DMSO controls at 26, 30 and 38h post invasion. P values are from Fishers exact tests, FDR from Benjamini Hochberg. Nested terms are redundant.

Supplementary table 15 Genes closest to summits of MSPC consensus peaks of ChIP Pf H2B.Z/input in Pfgcn5:ΔBRD.

Supplementary table 16 Genes closest to summits of MSPC consensus peaks of ChIP Pf H2B.Zac/input in Pfgcn5:ΔBRD.

Supplementary table 17 Genes closest to summits of MSPC consensus peaks of ChIP Pf H2B.Z/input in NF54::diCre-(Pfgcn5:loxP) DMSO treated controls.

Supplementary table 18 Genes closest to summits of MSPC consensus peaks of ChIP Pf H2B.Zac/input in NF54::diCre-(Pfgcn5:loxP) DMSO treated controls.

Suppl fig S1A)

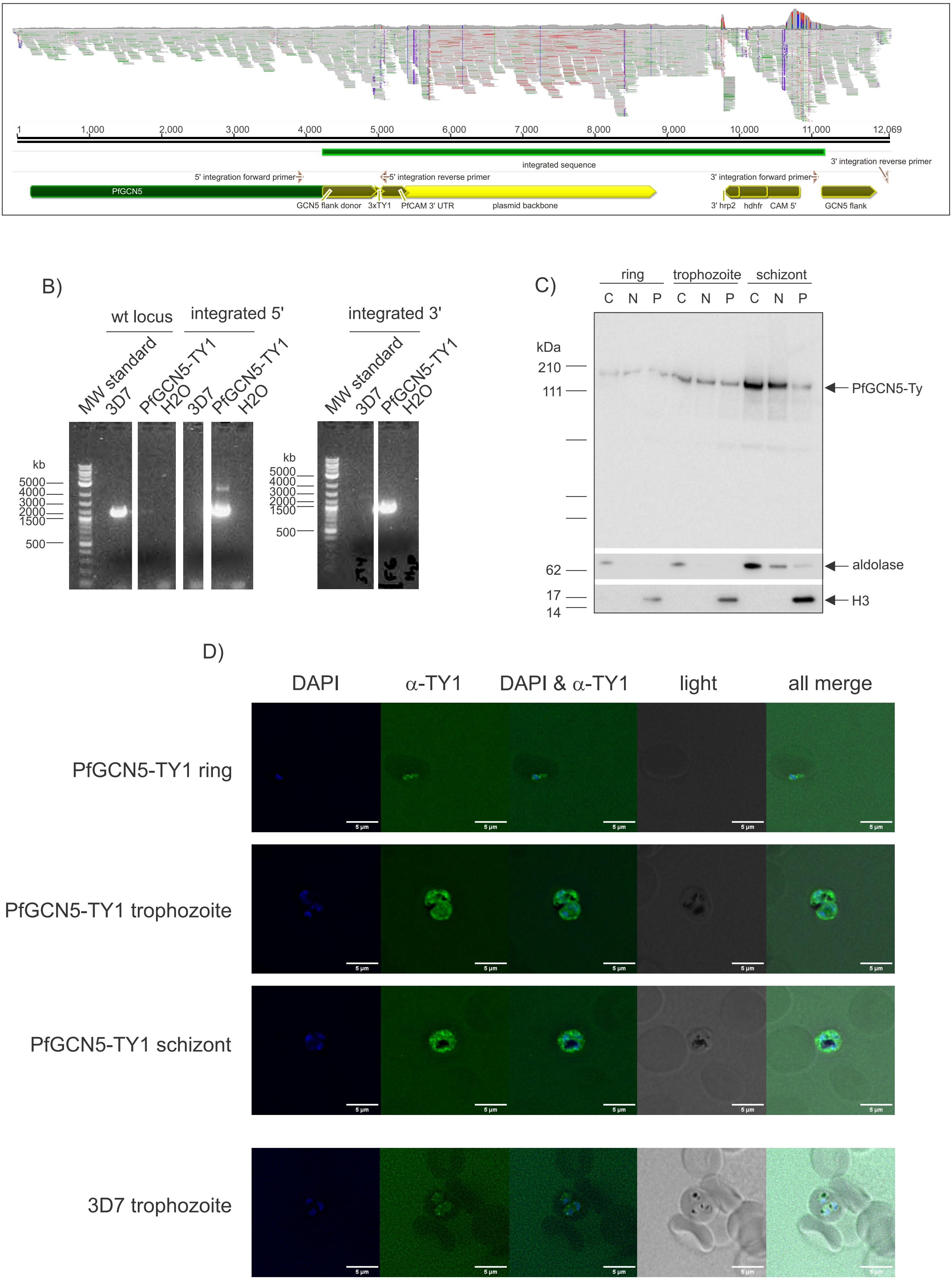

Supplementary Figure S1 Integration of 3 TY1 epitope tags at the 3’ end of PfGCN5 and cellular localisation of PfGCN5::TY1. A) A single cross-over strategy was used to integrate the plasmid pTgcn5:TY1 containing 3xTY1 epitope tags and a human dihydrofolate reductase selectable cassette at the 3’ end of *Pf*GCN5. Successful integration was confirmed by sequencing and by B) PCR using primers that amplified products spanning from the genome to the integrated sequences at both ends of the integration. All products were run on the same gel but lanes containing unrelated PCR products have been excised from the figure. The positions of the primers are also indicated in the diagram in panel A. C) Immunoblots of 3D7-(Pfgcn5::TY1) parasites ring stage (<16 hpi), trophozoite (24-34 hpi) and schizont (38-44 hpi) stage cytoplasmic (C), soluble nuclear (N) and insoluble nuclear (P) fractions probed with anti-TY1, anti-aldolase (cytoplasmic control) and anti-H3 (insoluble nuclear control). D) Indirect immunofluorescence with deconvolution of paraformaldehyde/ glutaraldehyde fixed 3D7-(Pfgcn5::TY1), and 3D7 parasites as controls for non-specific staining, probed with mouse anti-TY1 and then rabbit anti-mouse IgG. DNA was stained with DAPI.

Suppl fig S2

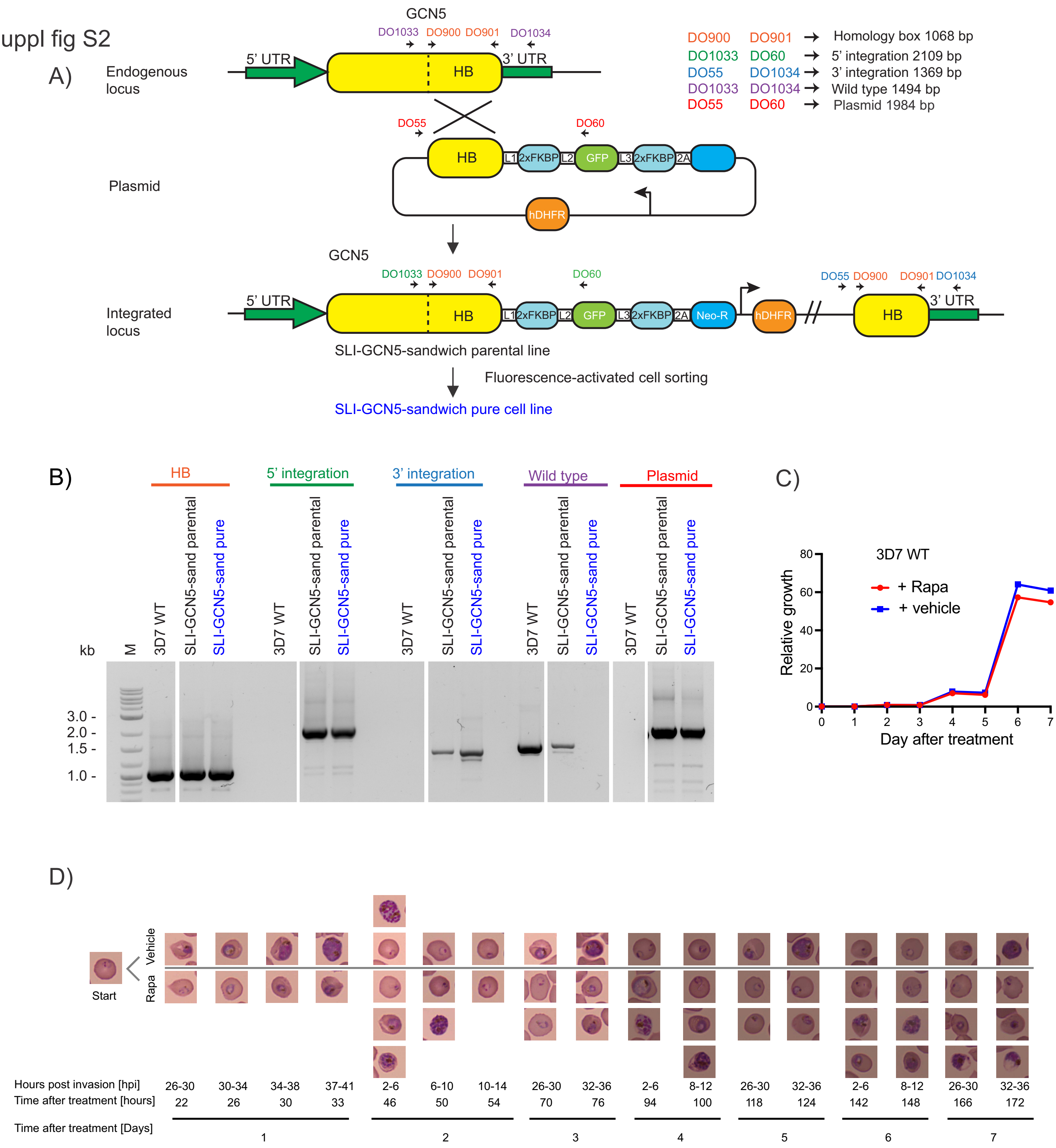
A)
GCN5
DO1033 DO900 DO901 DO1034
5' UTR
3' UTR
HB
Endogenous locus
DO900 DO901 → Homology box 1068 bp
DO1033 DO60 → 5' integration 2109 bp
DO55 DO1034 → 3' integration 1369 bp
DO1033 DO1034 → Wild type 1494 bp
DO55 DO60 → Plasmid 1984 bp
DO55
DO60
Plasmid
L1 2xFKBP L2 GFP L3 2xFKBP 2A
hDHFR
GCN5
DO1033 DO900 DO901
DO60
DO55 DO900 DO901 DO1034
Integrated locus
Neo-R
SLI-GCN5-sandwich parental line
Fluorescence-activated cell sorting
SLI-GCN5-sandwich pure cell line
B)
HB
5' integration
3' integration
Wild type
Plasmid
kb
M
3D7 WT
SLI-GCN5-sand parental
SLI-GCN5-sand pure
3.0 -
2.0 -
1.5 -
1.0 -
C)
3D7 WT
+ Rapa
+ vehicle
Relative growth
Day after treatment
D)
Vehicle
Rapa
Start
Hours post invasion [hpi]
Time after treatment [hours]
Time after treatment [Days]
26-30 30-34 34-38 37-41 2-6 6-10 10-14 26-30 32-36 2-6 8-12 26-30 32-36 2-6 8-12 26-30 32-36
22 26 30 33 46 50 54 70 76 94 100 118 124 142 148 166 172
1 2 3 4 5 6 7

Supplementary Figure S2 Creation and characterisation of 3D7-(Pfgcn5::Fkbp::Gfp) parasites used for knock-sideways. A, B) Integration of *Pf*GCN5-FKBP-GFP cassette for live microscopy and knock sideways. PCR confirmed integration of the cassette containing 2 FKBP dimerization domains followed by GFP and then two more FKBP dimerization domains, then the 2A skip peptide and Neomycin resistance gene and human *dhfr* cassette used for Selection Linked Integration. A) The PCR primers used are indicated on the legend to the right and on the endogenous and integrated loci and plasmid diagrams. B) Agarose gel electrophoresis of the amplified products. All products were run on the same gel but lanes containing unrelated PCR products have been excised from the figure. C) Rapalog used to induce knock sideways had no effect on wildtype 3D7 parasite growth. D) Growth following knock sideways (Rapa) and in controls (vehicle) was monitored by light microscopy of Giemsa stained, parasite infected erythrocytes.

Suppl fig S3

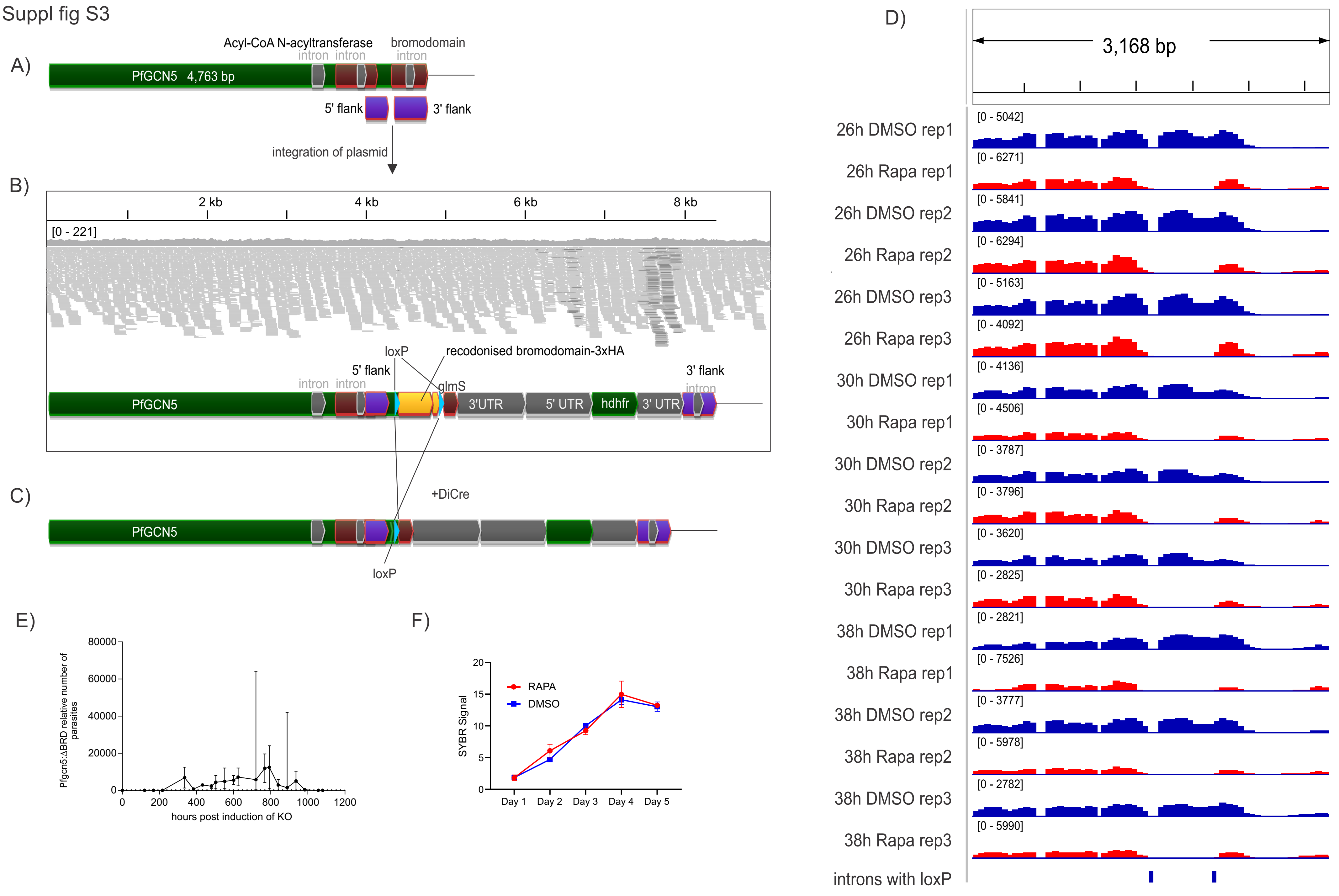

A)
Acyl-CoA N-acyltransferase
bromodomain
intron
intron
intron
PfGCN5 4,763 bp
5' flank
3' flank
integration of plasmid
B)
2 kb
4 kb
6 kb
8 kb
[0 - 221]
loxP
recodonised bromodomain-3xHA
5' flank
3' flank
intron
intron
glmS
intron
PfGCN5
3'UTR
5' UTR
hdhfr
3' UTR
C)
+DiCre
PfGCN5
loxP
D)
3,168 bp
26h DMSO rep1
[0 - 5042]
26h Rapa rep1
[0 - 6271]
26h DMSO rep2
[0 - 5841]
26h Rapa rep2
[0 - 6294]
26h DMSO rep3
[0 - 5163]
26h Rapa rep3
[0 - 4092]
30h DMSO rep1
[0 - 4136]
30h Rapa rep1
[0 - 4506]
30h DMSO rep2
[0 - 3787]
30h Rapa rep2
[0 - 3796]
30h DMSO rep3
[0 - 3620]
30h Rapa rep3
[0 - 2825]
38h DMSO rep1
[0 - 2821]
38h Rapa rep1
[0 - 7526]
38h DMSO rep2
[0 - 3777]
38h Rapa rep2
[0 - 5978]
38h DMSO rep3
[0 - 2782]
38h Rapa rep3
[0 - 5990]
introns with loxP
E)
Pfgcn5:ΔBRD relative number of parasites
80000
60000
40000
20000
0
0
200
400
600
800
1000
1200
hours post induction of KO
F)
SYBR Signal
20
15
10
5
0
RAPA
DMSO
Day 1
Day 2
Day 3
Day 4
Day 5

Supplementary Figure S3 Integration of a rapamycin inducible PfGCN5 bromodomain knockout cassette and its complete excision by Di-Cre inhibits growth. A) Diagram of wildtype *Pf*GCN5 with acetyltransferase domain, bromodomain and position of flank sequences used for homologous recombination to integrate recombinant sequences from donor plasmid. B) DNAseq reads mapped to the recombinant *Pf*GCN5 locus following integration of plasmid sequences with diagram of the recombinant locus below. C) Diagram of the locus following excision of floxed, recodonised, bromodomain. D) RNAseq readcounts mapped to the integrated, recombinant *Pf*GCN5 locus at multiple times following induction of *Pf*GCN5 excision following treatment with rapamycin or following treatment of controls with DMSO. Blue bars at the bottom indicate the positions of the loxP sites in the artificial introns. E) *Pfgcn5*:ΔBRD parasites grown continuously until death, relative growth is % parasitemia multiplied by culturing dilutions. Shown are triplicate (1-168, 552, 720, 888, 1056, 1080 hours post induction) and duplicate (216-504, 600, 624, 768-840, 936, 984 hours post induction) medians +/- range. F) NF54::DiCre parasites were grown in the presence of rapamycin or DMSO vehicle only for five days and growth analysed by incorporation of SYBR Green I into DNA mean ± SD.

Suppl Fig S4

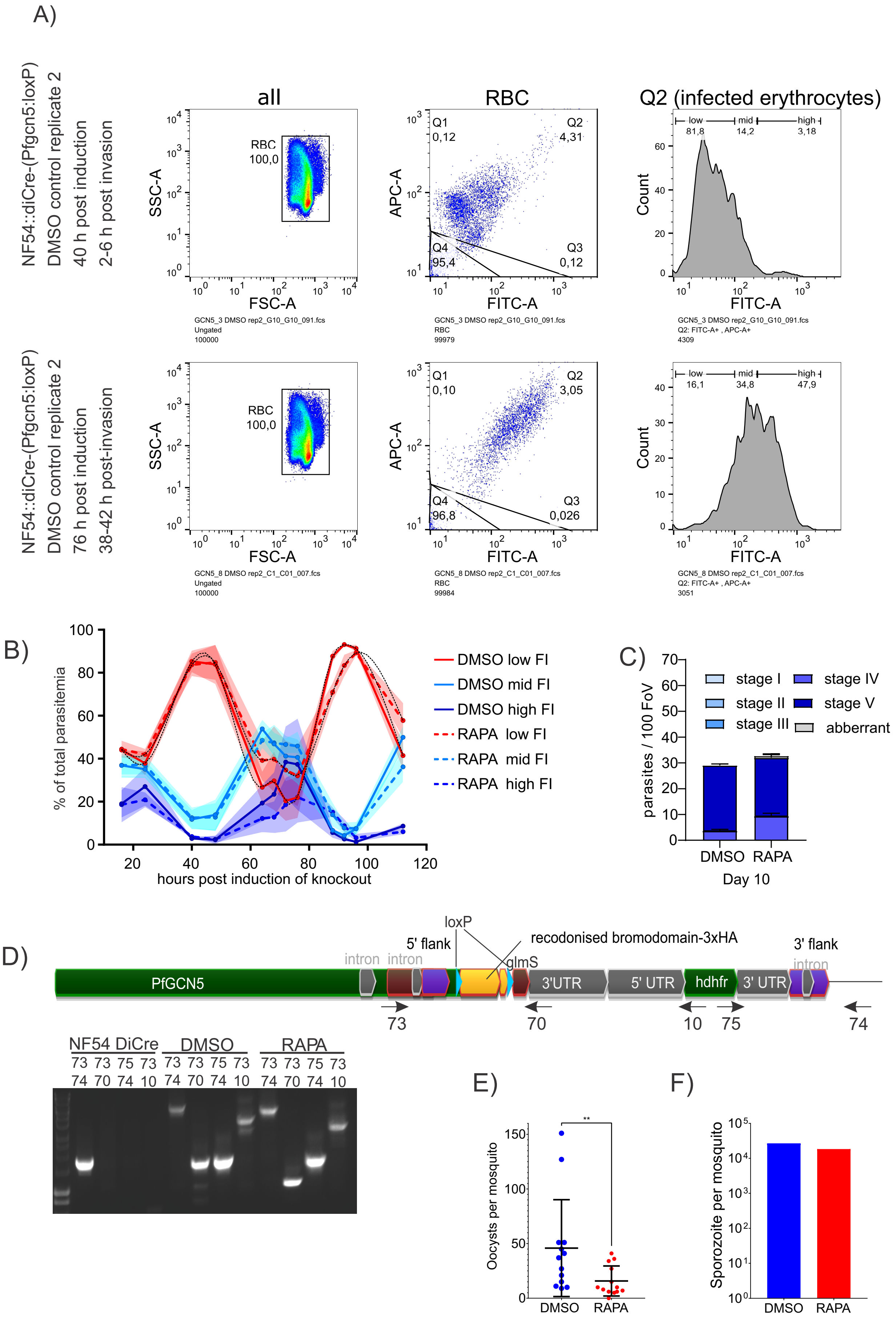
A)
NF54::diCre-(Pfgcn5:loxP)
DMSO control replicate 2
40 h post induction
2-6 h post invasion
all
RBC
Q2 (infected erythrocytes)
RBC 100,0
SSC-A
FSC-A
Q1 0,12
Q2 4,31
Q4 95,4
Q3 0,12
APC-A
FITC-A
low 81,8
mid 14,2
high 3,18
Count
GCN5_3 DMSO rep2_G10_G10_091.fcs
Ungated
100000
RBC
99979
Q2: FITC-A+ , APC-A+
4309
NF54::diCre-(Pfgcn5:loxP)
DMSO control replicate 2
76 h post induction
38-42 h post-invasion
RBC 100,0
Q1 0,10
Q2 3,05
Q4 96,8
Q3 0,026
low 16,1
mid 34,8
high 47,9
GCN5_8 DMSO rep2_C1_C01_007.fcs
Ungated
100000
RBC
99984
Q2: FITC-A+ , APC-A+
3051
B)
% of total parasitemia
hours post induction of knockout
DMSO low FI
DMSO mid FI
DMSO high FI
RAPA low FI
RAPA mid FI
RAPA high FI
C)
parasites / 100 FoV
stage I
stage II
stage III
stage IV
stage V
abberrant
DMSO
RAPA
Day 10
D)
loxP
5' flank
recodonised bromodomain-3xHA
3' flank
intron
intron
glmS
intron
PfGCN5
3'UTR
5' UTR
hdhfr
3' UTR
73
70
10
75
74
NF54 DiCre
DMSO
RAPA
73 73 75 73
74 70 74 10
E)
**
Oocysts per mosquito
DMSO
RAPA
F)
Sporozoite per mosquito
DMSO
RAPA

Supplementary Figure S4. Knockout of the *Pf*GCN5 bromodomain causes developmental defects. A) Gating strategy for flow cytometry analysis. Infected erythrocytes were defined as SYTO61 (APC channel) and Thiazole Orange (FITC channel) double positive cells (Q2), and populations of low, mid and high fluorescence intensity were quantified, representing progressive stages of parasite development. Two samples are shown as representative examples for a predominantly ring stage (upper row) or schizont stage culture (lower row). B) Cytometry of different developmental stages gated by cytometry of nucleic acid fluorescent staining. Point to point lines plotted for means ± SD (shading) of the percentages of parasites that were gated as low Fluorescence intensity (FI), mid FI or high FI across the 112h cytometry assay following induction of knockout of the PfGCN5 BRD (RAPA) or treatment of controls with DMSO. Black lines are cubic spline curves fitted to the low FI gated population. C) Proportions of gametocyte stages in Giemsa smears from DMSO and RAPA treated NF54::DiCre cultures on day 10 of gametocyte maturation (n=3). FoV: field of view. D) PCR confirms excision of loxP flanked *Pf*GCN5 BRD after rapamycin treatment (RAPA) on the seventh day after induction of gametocytogenesis compared to DMSO treated control and NF54::DiCre gametocytes. Primer positions are indicated on diagram of recombinant locus above the photo of the gel. E) Number of oocysts per mosquito produced by NF54::DiCre parasites treated with rapamycin (RAPA) or DMSO (oocysts numbers pooled from three experiments, **$p<0.01$ Mann-Whitney U test). F) Number of sporozoites per mosquito produced by NF54::DiCre parasites treated with rapamycin (RAPA) or DMSO.

Suppl Fig S5

A)

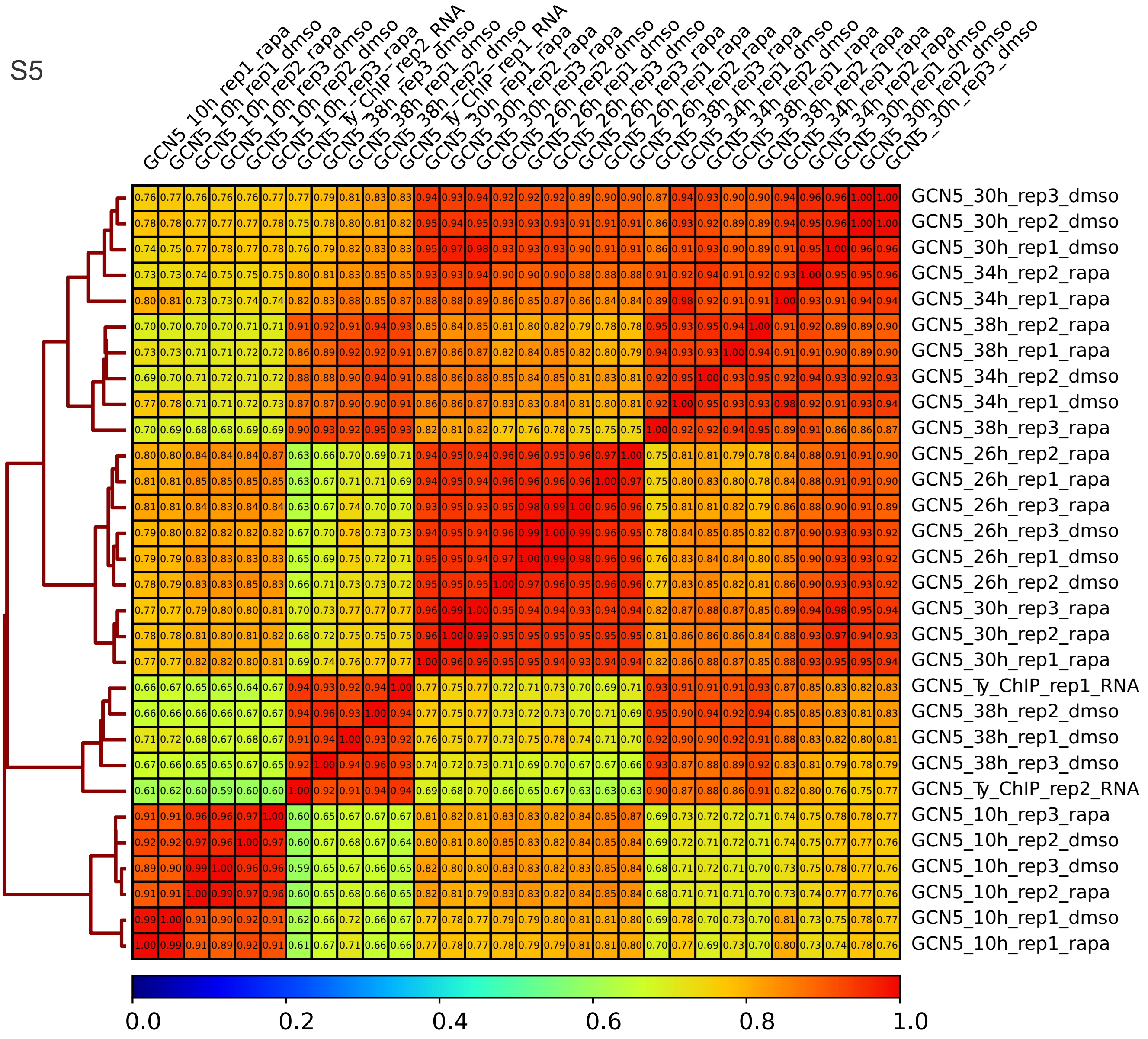

B)

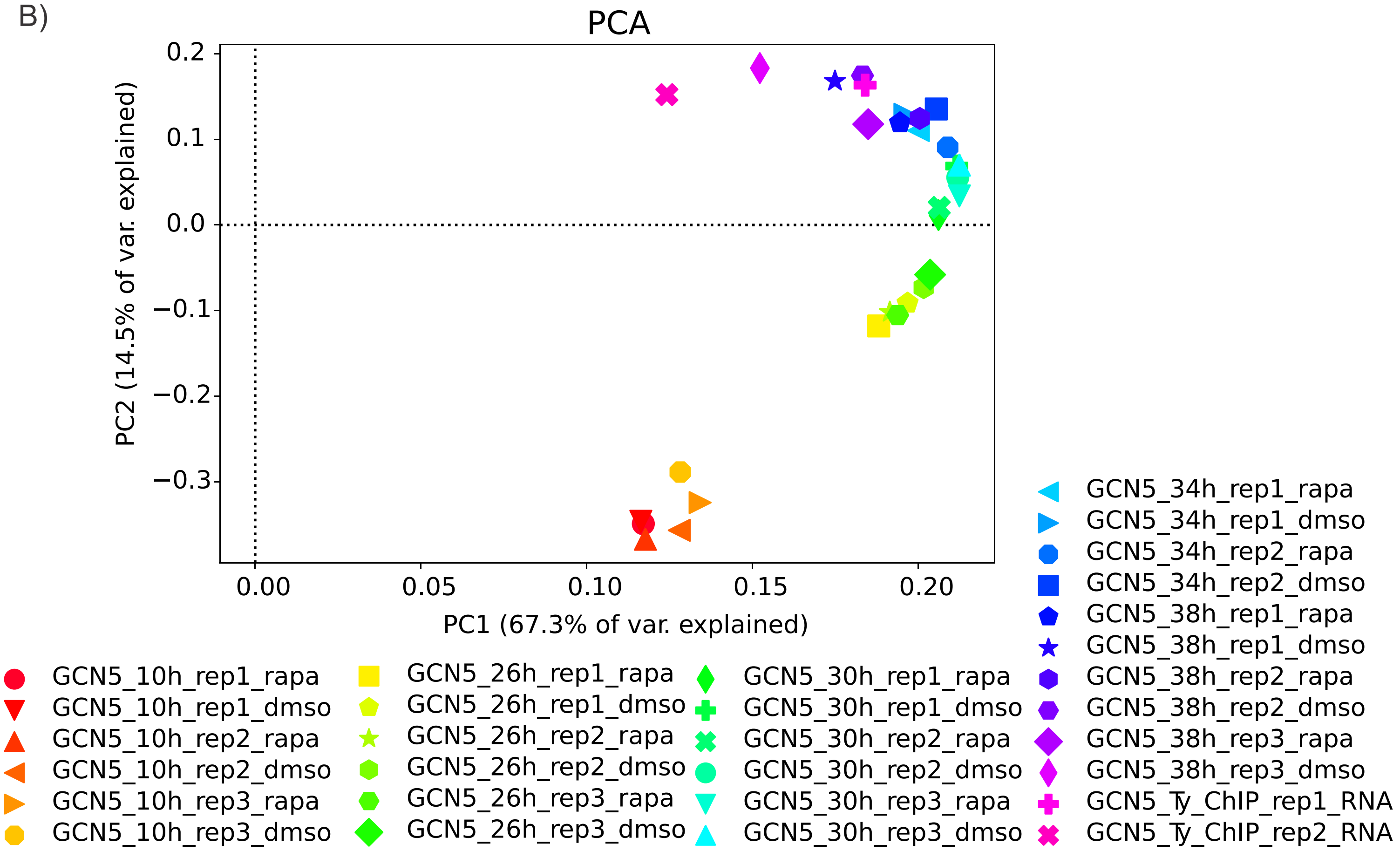

Supplementary Figure S5. Similarities between RNAseq samples. RNAseq replicates of *Pfgcn5*:ΔBRD and matched DMSO treated controls at 10, 26, 30, 34 and 38 hpi in the second cycle following deletion of the *Pf*GCN5 BRD as well as the matched RNAseq samples from the 38 hpi 3D7-(*Pf*GCN5Pfgcn5-::TY1) parasites used for ChIPseq of *Pf*GCN5::TY1 were compared by A) Spearman correlations and B) Principal Component Analysis.

Suppl fig S6

A)

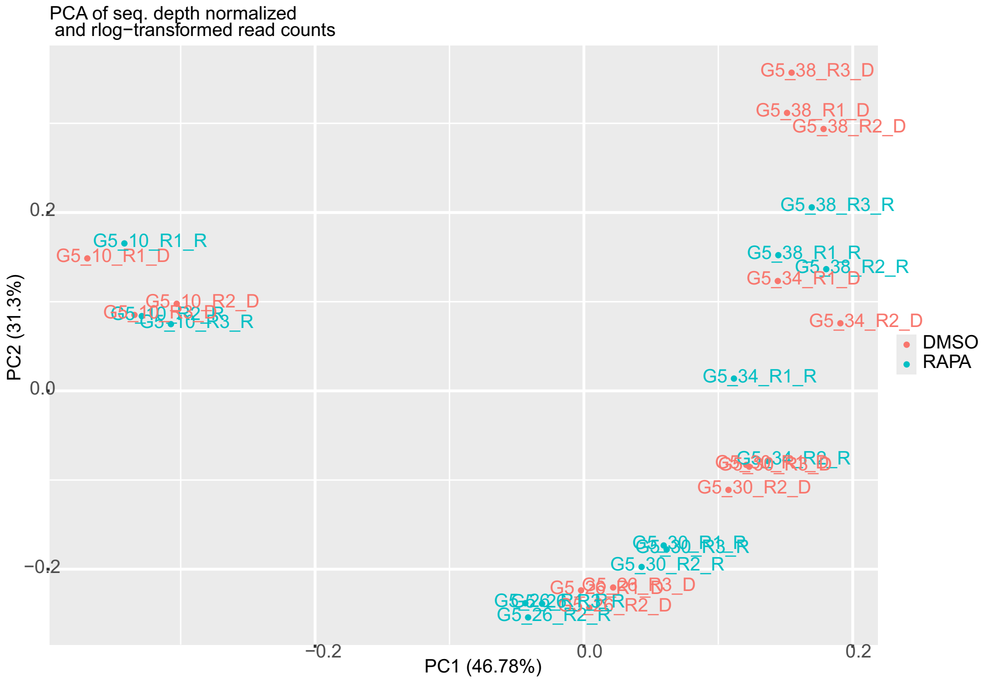

B)

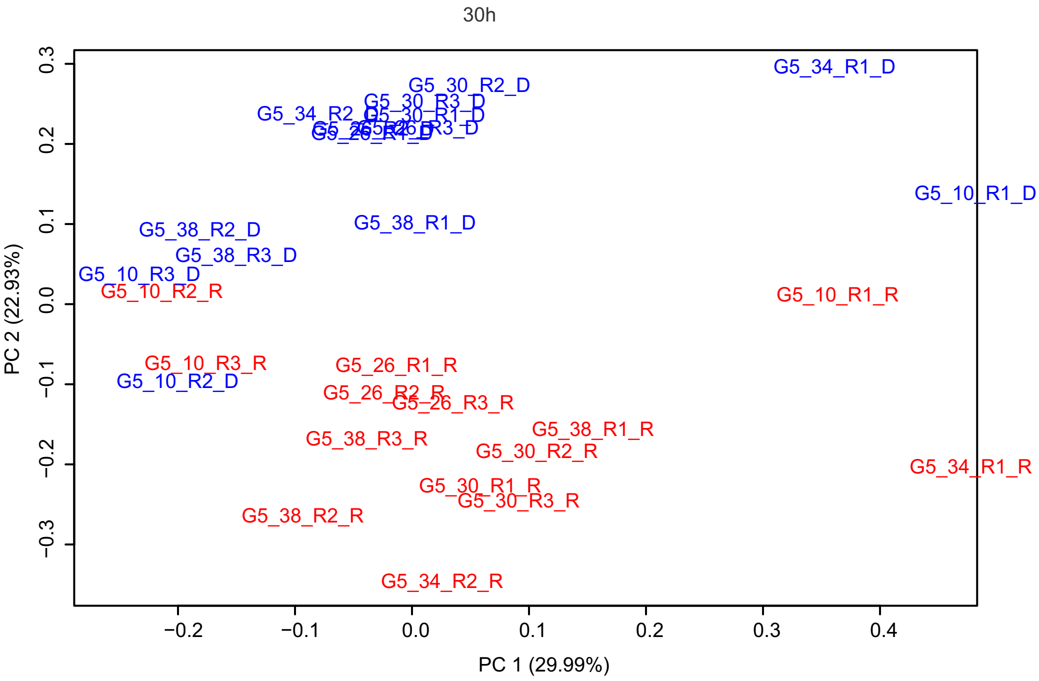

C)

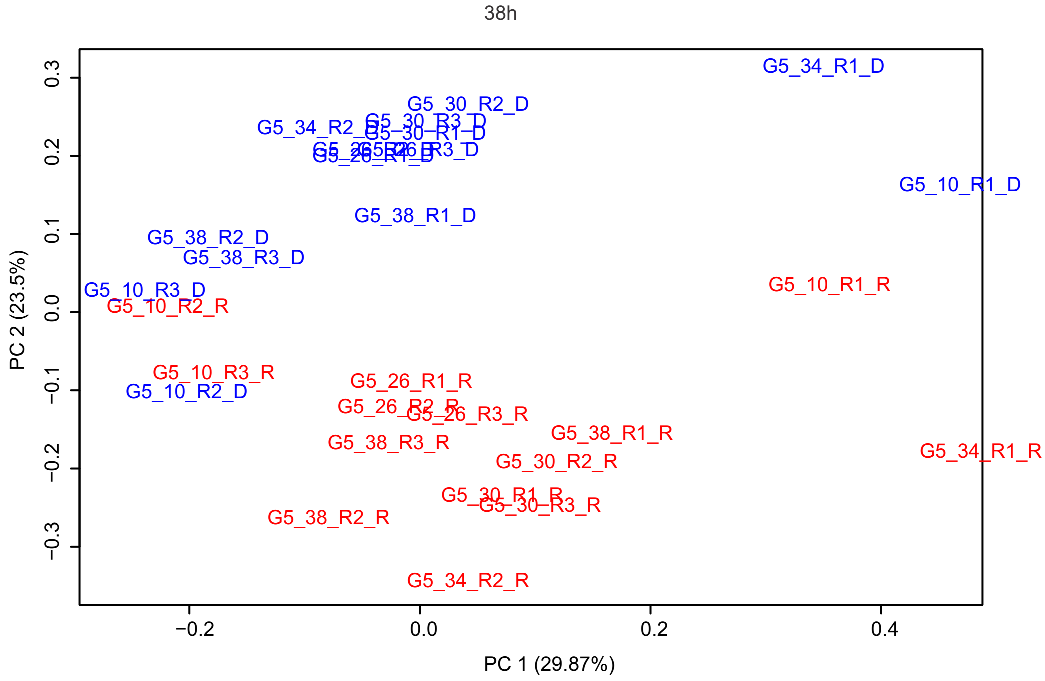

Supplementary Figure S6. Removal of unwanted factors of variation from 30 hpi and 38 hpi Differential Gene Expression Analysis. A) PCA plot of Deseq2 size factor normalised and r-log transformed readcounts for all *Pfgcn5*:ΔBRD and control samples from RNAseq timecourse. Samples are named G5(GCN5)_10, 26, 30, 34 or 38 (hpi)_R1, 2, 3(replicate 1, 2 or 3)_R (rapamycin induced knockout) or_D (DMSO control). B) PCA plot of Deseq2 size factor normalised and r-log transformed readcounts after correcting for 2 factors of variation using 30 hpi and 26 hpi DMSO controls all as 30 hpi DMSO replicates. Sample naming as for panel A. C) PCA plot of Deseq2 size factor normalised and r-log transformed readcounts after correcting for 2 factors of variation using 38 hpi and 34 hpi DMSO controls all as 38 hpi DMSO replicates. Sample naming as for panel A.

Suppl fig S7

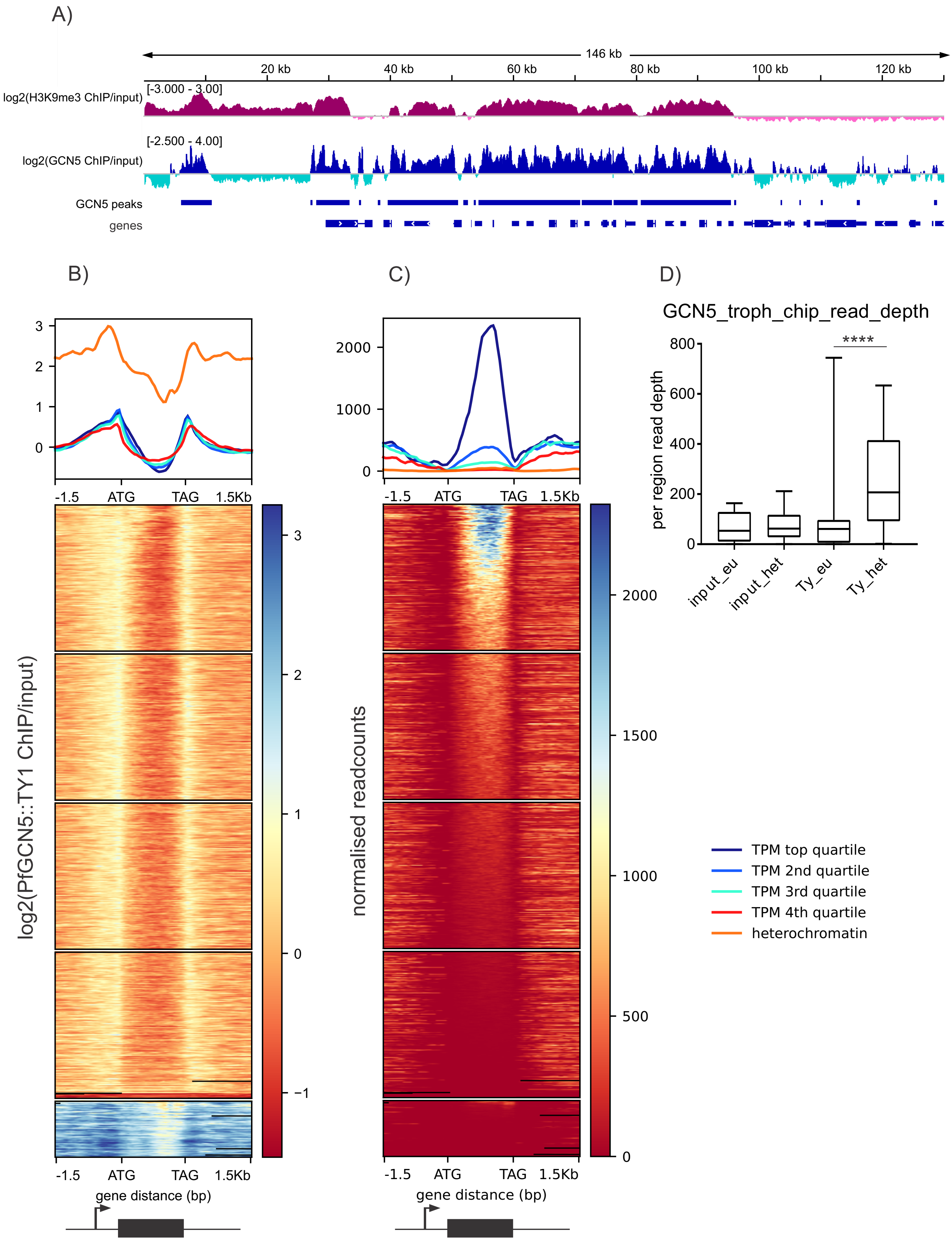

A)
146 kb
20 kb
40 kb
60 kb
80 kb
100 kb
120 kb
log2(H3K9me3 ChIP/input)
[-3.000 - 3.00]
log2(GCN5 ChIP/input)
[-2.500 - 4.00]
GCN5 peaks
genes
B)
C)
D)
GCN5_troph_chip_read_depth
log2(PfGCN5::TY1 ChIP/input)
normalised readcounts
per region read depth
-1.5
ATG
TAG
1.5Kb
gene distance (bp)
****
input_eu
input_het
Ty_eu
Ty_het
TPM top quartile
TPM 2nd quartile
TPM 3rd quartile
TPM 4th quartile
heterochromatin

Supplementary Figure S7 Uniquely mapped reads confirm heterochromatic enrichment of PfGCN5. A) IGV projection of the left end of chromosome 1 showing log2(ChIP/input) for H3K9me3 and *Pf*GCN5 for primary reads mapped by Bowtie2. B-C) Native ChIP enrichment of *Pf*GCN5::TY in heterochromatin was not due to an artefact of lower coverage of input in heterochromatin than in euchromatin that was reported for cross-linked ChIP (44). Reads from two anti-TY1 ChIPseq replicates of 3D7-(Pfgcn5::TY1) were mapped using the -c 1 argument of BWA to filter uniquely mapped reads only. B) The average log2Chip/input of the two anti-TY1 3D7-(Pfgcn5::TY1) ChIPseq replicates. C) Average normalised readcounts from two 3D7-(Pfgcn5::TY1) RNAseq replicates matched to the anti-TY1 ChIPseq samples. Both heatmaps are ranked by descending order of gene expression. Average line plots of the heatmap data are on top of the heatmaps. D) Per region read depth of bowtie2 mapped primary alignments compared between euchromatin and H3K9me3 enriched heterochromatin for input and GCN5-Ty native ChIP, median, IQR and range, Mann Whitney U test ****$p<0.0001$.

Suppl fig S8

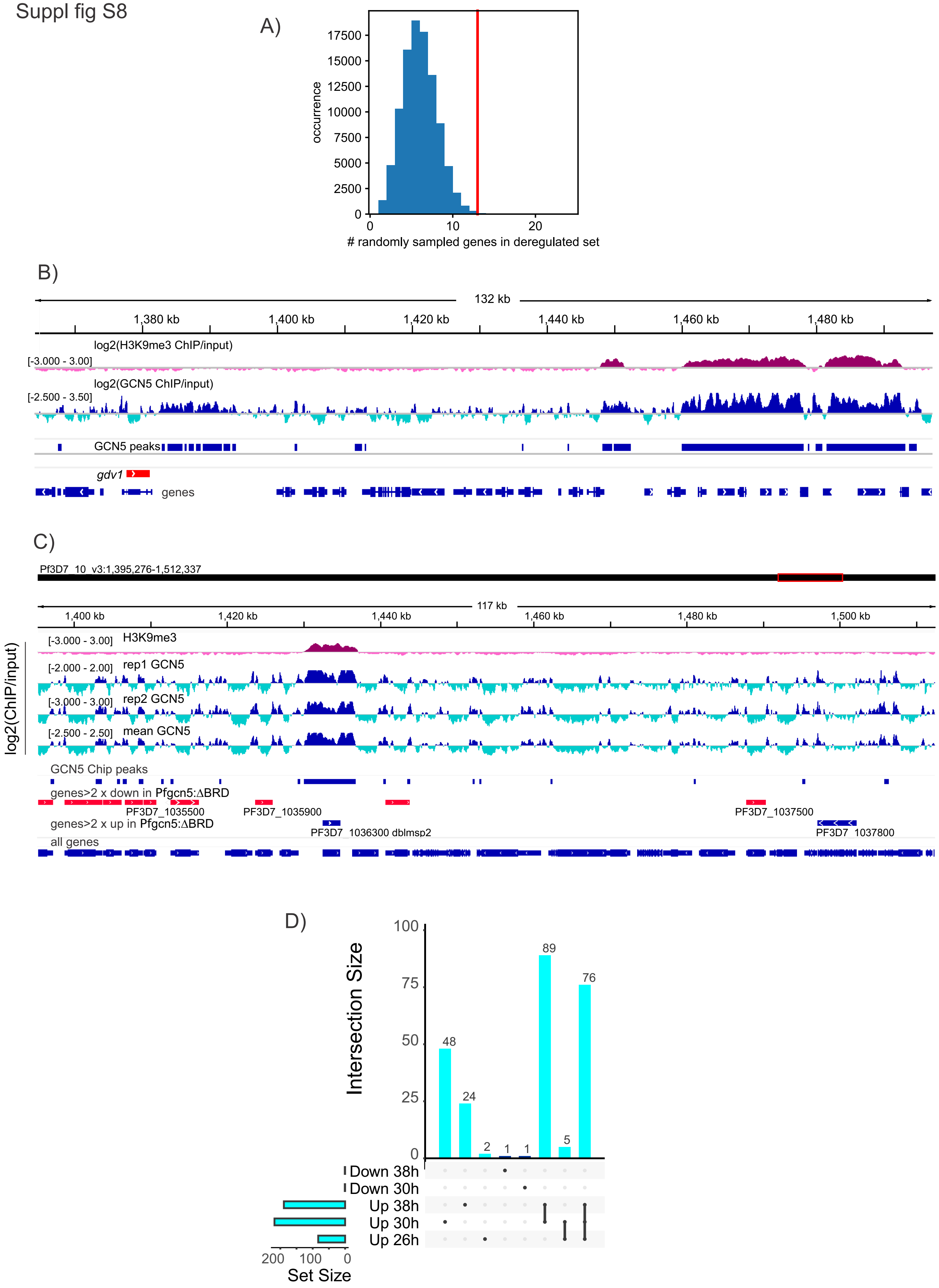
A)
occurrence
17500
15000
12500
10000
7500
5000
2500
0
0
10
20
randomly sampled genes in deregulated set
B)
132 kb
1,380 kb
1,400 kb
1,420 kb
1,440 kb
1,460 kb
1,480 kb
log2(H3K9me3 ChIP/input)
[-3.000 - 3.00]
log2(GCN5 ChIP/input)
[-2.500 - 3.50]
GCN5 peaks
gdv1
genes
C)
Pf3D7_10_v3:1,395,276-1,512,337
117 kb
1,400 kb
1,420 kb
1,440 kb
1,460 kb
1,480 kb
1,500 kb
log2(ChIP/input)
[-3.000 - 3.00] H3K9me3
[-2.000 - 2.00] rep1 GCN5
[-3.000 - 3.00] rep2 GCN5
[-2.500 - 2.50] mean GCN5
GCN5 Chip peaks
genes>2 x down in Pfgcn5:ΔBRD
PF3D7_1035500
PF3D7_1035900
PF3D7_1037500
genes>2 x up in Pfgcn5:ΔBRD
PF3D7_1036300 dblmsp2
PF3D7_1037800
all genes
D)
Intersection Size
100
75
50
25
0
48
24
2
1
1
89
5
76
Down 38h
Down 30h
Up 38h
Up 30h
Up 26h
200
100
0
Set Size

Supplementary Figure S8. Characteristics of genesets deregulated in *Pfgcn5*:ΔBRD. A) Histogram showing the number of genes belonging to the set of 2927 genes that were deregulated in *Pfgcn5*:ΔBRD (DESeq2 adj $p<0.05$ for a randomly sampled set of 25 genes. 100,000 random samples were analysed. The red line indicates 13 which was the number of genes that were deregulated within the set of 25 genes that were present in PfGCN5 broad peaks. B) IGV projection of the *gdv1* gene and surrounding sequence showing log2(ChIP/input) for H3K9me3 and *Pf*GCN5. C) IGV projection of the *dblmsp2* gene and surrounding sequence showing log2(ChIP/input) for *Pf*GCN5 and H3K9me3, the consensus PfGCN5 ChIPseq peaks and the position of genes more than 2 fold deregulated at 38 hpi in *Pfgcn5*:ΔBRD. D) UpSet plot showing intersections between deregulated heterochromatic genesets at different timepoints in *Pfgcn5*:ΔBRD parasites.

Suppl fig S9

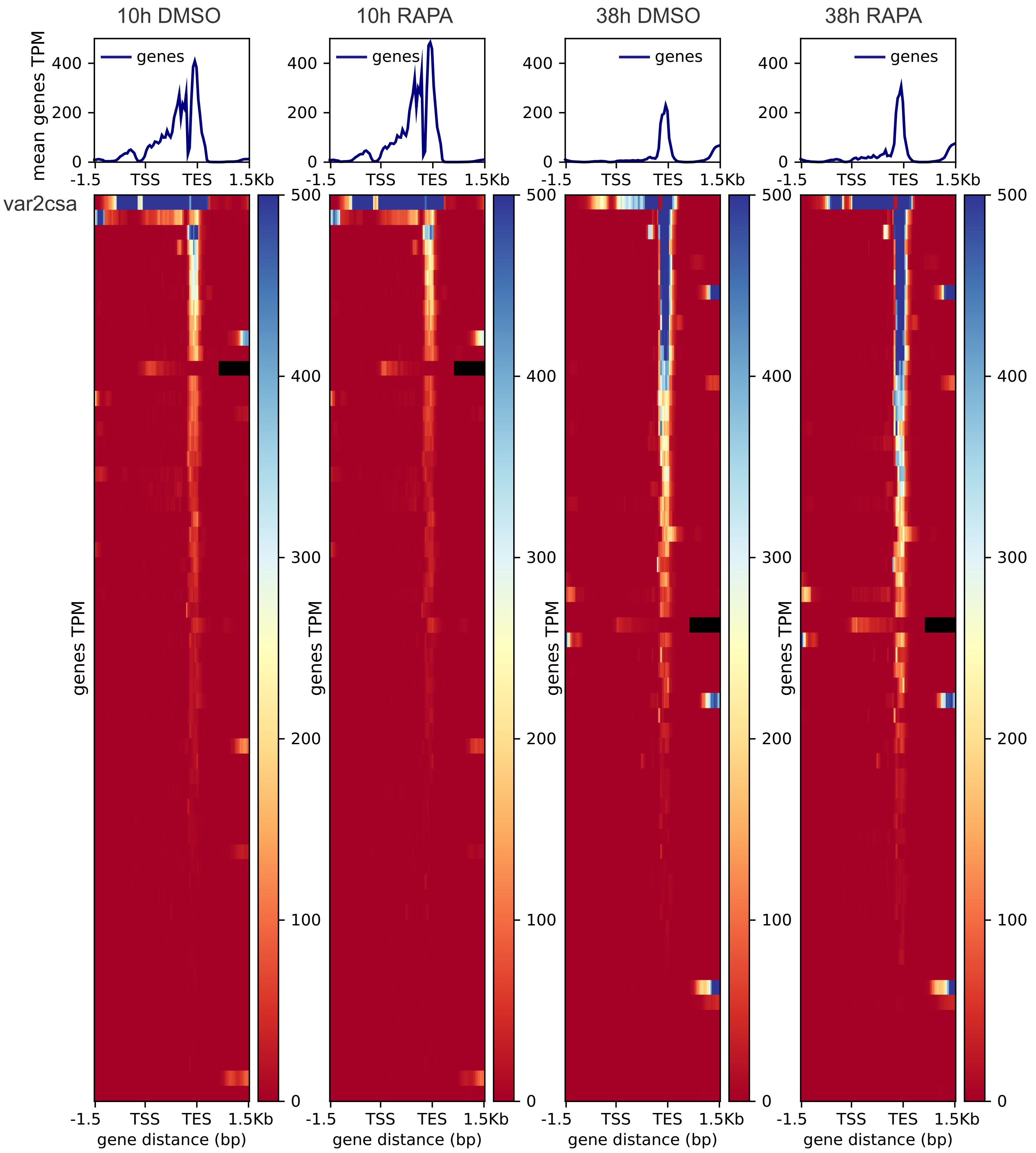

10h DMSO
10h RAPA
38h DMSO
38h RAPA
genes
mean genes TPM
400
200
0
-1.5
TSS
TES
1.5Kb
var2csa
genes TPM
500
400
300
200
100
0
gene distance (bp)

Supplementary Figure S9 Expression (TPM) of all *var* genes from 1.5 kb upstream of the start codon to 1.5 kb downstream of the stop codon in NF54::diCre-(Pfgcn5:loxP) parasites at 10 h and 38 h post treatment with DMSO or rapamycin. Heatmaps are ranked by descending order of gene expression in DMSO controls.

Suppl fig S10

3D7-(Pfgcn5::Fkbp::Gfp) knock-sideways

A)

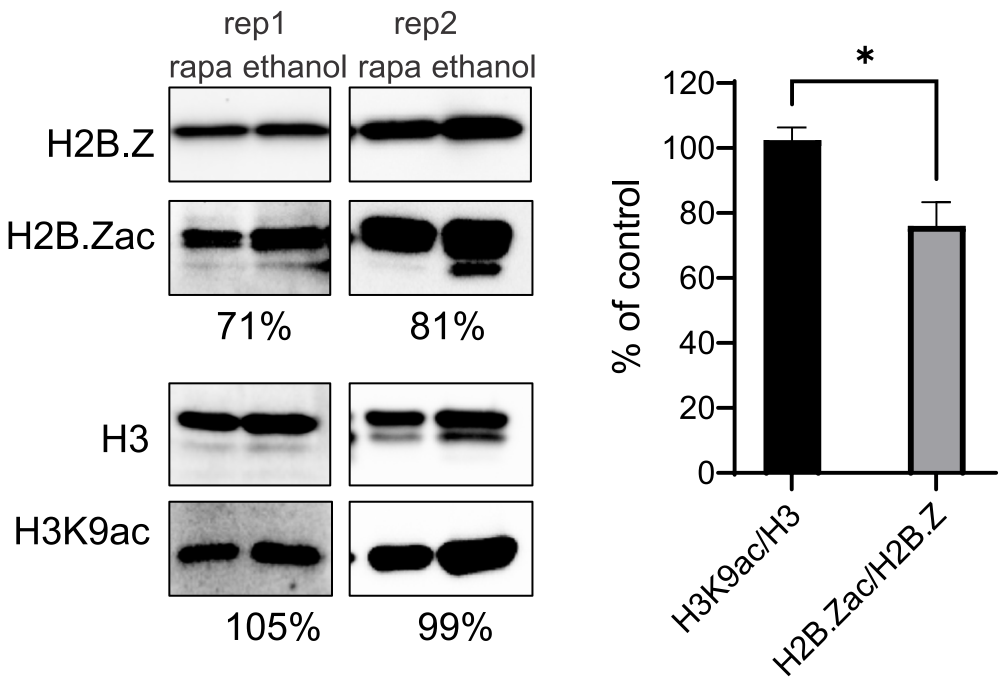

B)

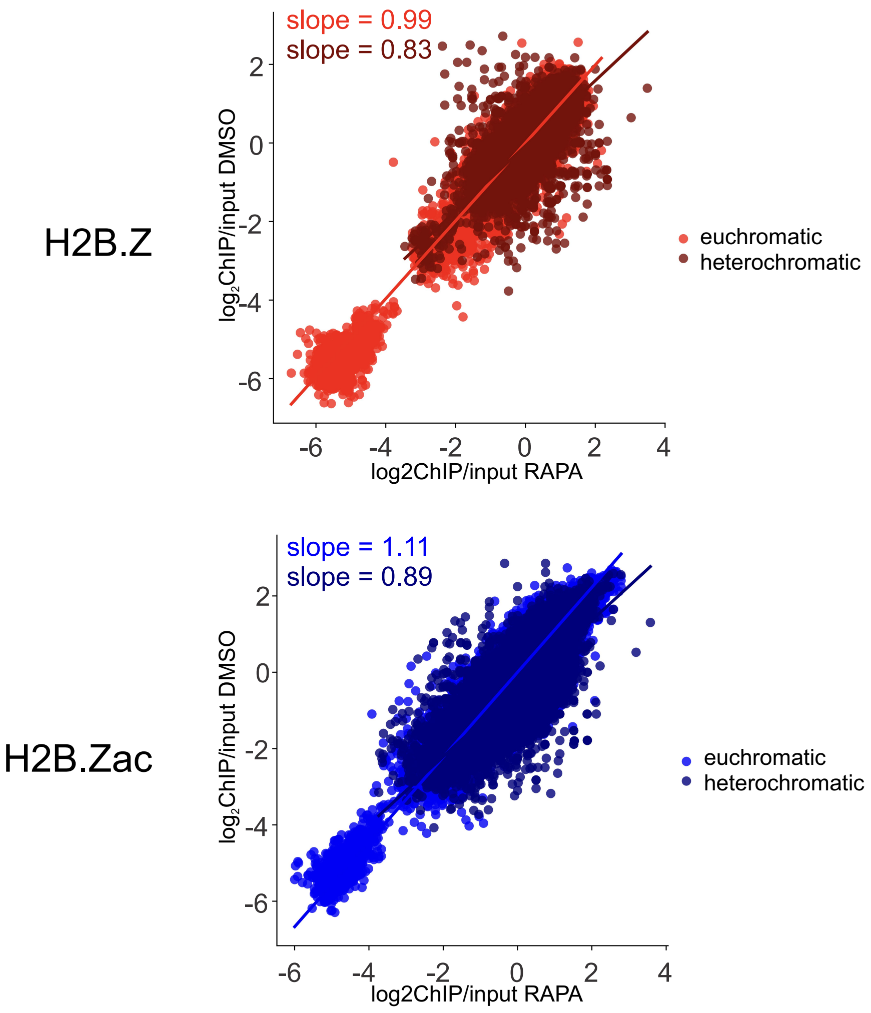

Supplementary Figure S10 Impact of PfGCN5 BRD deletion or depletion on histone acetylation. A) PfGCN5::FKBP::GFP was mislocalised to the plasma membrane in 3D7-(Pfgcn5::Fkbp::Gfp) parasites by treating with 6.2 nM rapalog or 1/40 ethanol vehicle control from invasion for 32 hours and then extracted proteins were separated by electrophoresis and probed with antibodies to H3 and H3K9ac or H2B.Z and H2B.Zac. The signal intensity was determined by densitometry and the ratio of acetylated/total histone was calculated and expressed in % relative to the ethanol treated control. Error bars represent SD of N=2 replicates, unpaired t-test, * $p<0.05$. B) Scatter plots of log2 ChIP/input enrichment levels in 50 bp bins across the genome. Slopes were calculated for heterochromatic and euchromatic genome regions (N=2).
